# Dissimilarity between living and dead benthic foraminiferal assemblages in the Aveiro Continental Shelf (Portugal)

**DOI:** 10.1371/journal.pone.0209066

**Published:** 2019-01-30

**Authors:** Maria Virgínia Alves Martins, Johann Hohenegger, Fabrizio Frontalini, João Manuel Alveirinho Dias, Mauro Cesar Geraldes, Fernando Rocha

**Affiliations:** 1 Departamento de Estratigrafia e Paleontologia, Faculdade de Geologia, Universidade do Estado do Rio de Janeiro, Rio de Janeiro, Brazil; 2 Departamento Geociências, GeoBioTec, Universidade de Aveiro, Aveiro, Portugal; 3 Institut für Paläontologie, Universität Wien, Wien, Austria; 4 Dipartimento di Scienze Pure e Applicate (DiSPeA), Università degli Studi di Urbino "Carlo Bo", Urbino, Italy; 5 CIMA, Centro de Investigação Marinha e Ambiental, Universidade do Algarve, Campus de Gambelas, Faro, Portugal; National Cheng Kung University, TAIWAN

## Abstract

This study compares living (LA) and dead (DA) benthic foraminiferal assemblages and identifies different factors that possibly cause differences in the distribution of both assemblages in the Aveiro Continental Shelf (Portugal). A total of 44 sediment samples was collected during summers of 1994 and 1995 along transects (east-west direction) and between 10 and 200 m water depth. Complex statistical analyses allow us to compare the abundance and composition of the LAs and DAs in function of depth, grain-size and total organic matter in all studied stations even in those where the numbers of individuals were rare in one or both assemblages. The highest densities and diversities of the LAs are found in the middle continental shelf on gravel deposits (coarse and very coarse sands) mostly due to the substrate stability, reduced deposition of fine sedimentary particles, availability of organic matter with high quality related to oceanic primary productivity likely induced by upwelling events, and oxygenated porewaters conditions. The DAs have, in general, higher densities and diversities than the LAs. In the outer continental shelf, the dissimilarity between both assemblages is higher due to the accumulation of tests, low dilution by sedimentary particles and scarcity of living foraminifera. Based on the comparison of LAs and DAs and considering the characteristics of the study area and the species ecology, it has been possible to understand the cause of temporal deviation between the LAs and DAs of benthic foraminifera. This deviation is much more pronounced in the inner shelf where the energy of the waves and the currents induce very dynamic sedimentary processes preventing the development of large LAs and the preservation of DAs. Some deviation also occurs in the middle shelf due to the seasonal loss of empty tests. The most well-preserved time-averaged DAs were found in the outer continental shelf.

## Introduction

The evaluation of environmental quality represents a priority, particularly in coastal and transitional marine settings where most of the anthropogenic activities are placed. Since historical time, human activities have modified and continuously affected these areas though their intensity has grown over the last centuries. The definition of baseline environmental conditions or the identification of pristine areas represent, therefore, a key to understand the effect of human activities [1]. Since fully pristine-undisturbed areas with similar environmental features to our target are difficult to find if existed [2], the investigation of the sedimentary record for defining baseline conditions represents a good alternative. Unfortunately, the comparison of dead assemblages (DAs) along the sedimentary records to the living counterpart (LAs) is not so straightforward as taphonomic processes including, for instance, differential transportation, destruction, and other postmortem process, occur [3]. Moreover, the mismatches between LAs and DAs assemblages are not only determined by natural process but also induced by anthropogenic impacts that further hinder the definition of reliable baseline conditions [1,3]. A lower level of fidelity between living-dead molluscan assemblages was, for instance, documented in areas with enhanced anthropogenic eutrophication [1,3]. or in narrow shelves [3] as new compositional states present in LAs are not yet captured by DAs. Under these circumstances, the development of new methodologies, the test of their applicability, the estimation of the discordance of LAs *vs*. DAs and the bias drivers are important milestone in both a paleontological and an actuopaleontological perspective. Specifically, the deviation of LAs-DAs might also represent a useful tool by which identifying the source of biases such as the environmental degradation and identifying areas suitable for paleoenvironmental and paleoecological reconstructions in both deep and recent time. This actualistic approach has been applied in transitional environments like estuaries and lagoons as well as more open shelf setting and based on mollusk, ostracods, scallop and benthic foraminifera [1–6]. Although the LAs are widely used to determine the small-scale variations, the DAs might also represent an important source of biological information such as the inventory of rare species and the estimation of anthropogenically shifted baselines [7].

Benthic foraminifera, single-celled organisms, are widely used as bioindicators in environmental biomonitoring in both oceanic [8–10] and in coastal transitional environments [11–18]. Unfortunately, only few investigations have been aimed to compare the living (LAs) and dead (DAs) benthic foraminiferal assemblages [5,6,19–22]. As LAs are more directly related to ecological and environmental conditions at the time of sampling [23], their use has been included in the FOBIMO protocol as mandatory recommendation [24].

This work aims to compare both DAs and LAs in terms of abundance, diversity and composition and in relation to depth, grain-size and Total Organic Matter (TOM) on the western Portuguese Continental Shelf (PCS), specifically in the Aveiro Continental Shelf (ACS) by using a set of statistical methods even where the density of specimens was scarce in one or both assemblages. It also seeks to understand the causes of the observed deviation between LAs and DAs of benthic foraminifera.

## Study area

**Fig 1:**
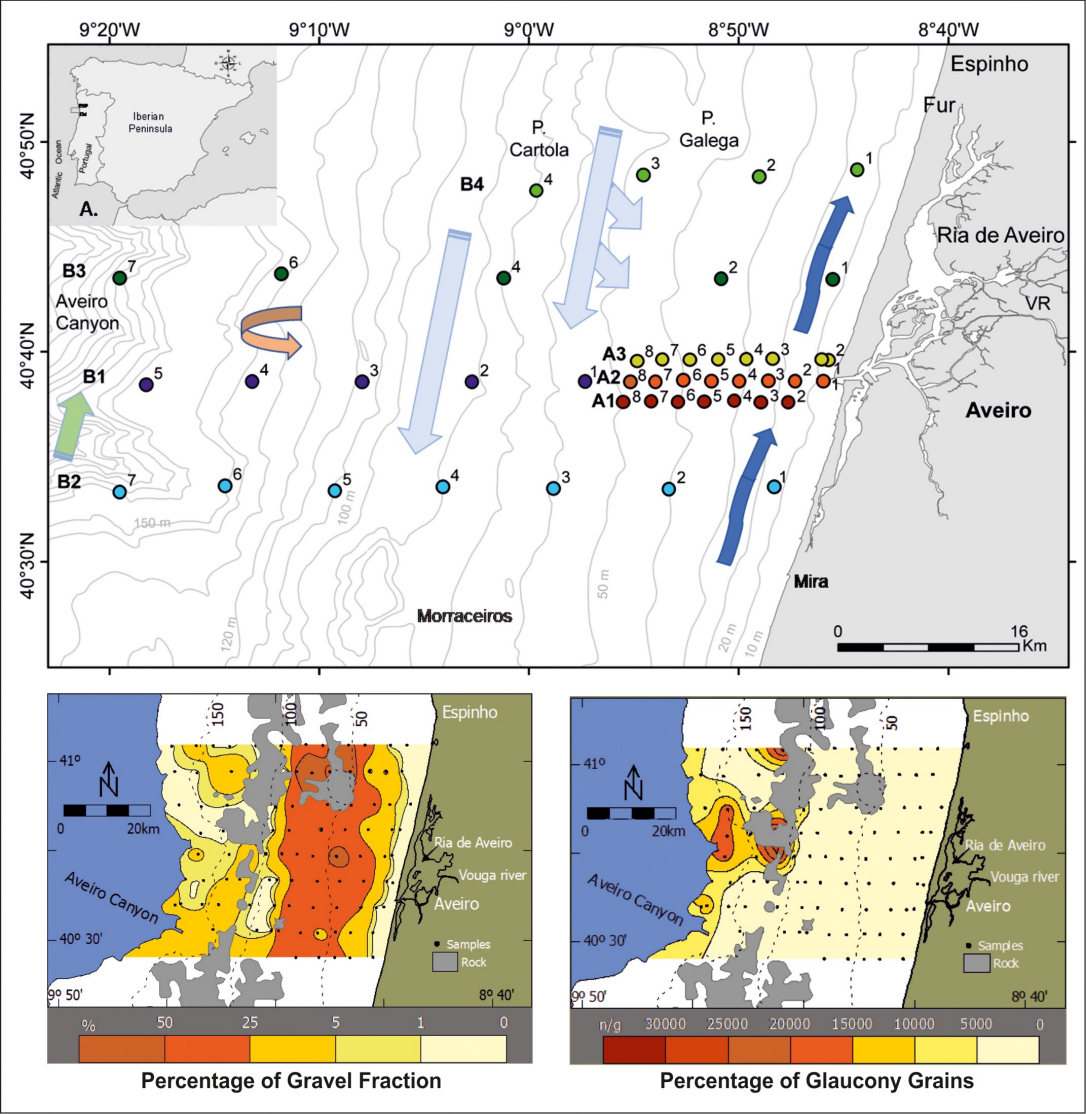
A. Study area (rectangle). B. Location of sample sites in transects with different colors offshore the Ria de Aveiro lagoon mouth. Legend: P. Cartola—Pontal da Cartola; P. Galega–Pontal da Galega; Fur–Furadouro; VR—Vouga River (adapted from Martins et al. [58]).

The study area is located in the northern sector of the PCS between Espinho and Cabo Mondego (Fig 1). It is included in the North Atlantic Province, characterized by annual average temperatures of about 15°C [25]. The meteorological conditions of the Portuguese coast are influenced by the high-pressure system of the Azores and, to a lesser extent, by the low-pressure center of Iceland [26]. The displacement of these two masses of air produces dry and stable weather in summer and rainy and unstable weather in winter [26]. In this region, the wind regime is characterized by the predominance of the NW and N winds direction. The S and SW winds are less frequent but have higher speeds. The most intense winds mainly occur from October to May, with the strongest winds (>50 km/h) in December and February [27]. The northern winds, of moderate intensity, occur mainly in summer and are generated by the Azores anticyclone and thermal depression in the Iberian Peninsula during this season. In winter, the wind regime presents greater variability in direction and intensity [26].

These meteorological conditions influence the oceanographic regime as well as the structure of the water masses on the continental shelf [28]. A superficial oceanic layer is distinguished. It comprises the layer of superficial mixture and the seasonal thermocline of variable thickness throughout the year due to the seasonality of the atmosphere-ocean fluxes (heating /cooling, mixture induced by the wind, precipitation /evaporation) and rivers’ flows [29]. Near the study area, at 40°N and 12°W, this layer may reach about 200 m depth [29]. The Eastern North Atlantic Central Water is placed below this thin superficial layer and is characterized by temperature and salinity values decreasing with depth [28]. It is constituted by two layers of different origins. A southern one, of subtropical origin, with higher temperature and salinity, is formed during winter along the Azores Front at the latitude of about 34°N-35°N [29]. The northern one of subpolar origin near 50°N with lower temperature and salinity, is formed by deep winter convection [29]. The surface circulation is predominantly northward off the Iberian Peninsula during autumn and winter [30–33]. As a result of the Azores Anticyclone migrating to the north and the weakening of the Icelandic Low-Pressure Nucleus in spring and summer, a prevailing regime of N and NW winds (northerly regime) is established that are favorable to upwelling [26,34]. This phenomenon constitutes one of the main processes that determine the oceanographic characteristics of the Portuguese coastal waters during summer [26]. Associated with the summer regime, the circulation pattern is characterized on the continental shelf by a surface runoff to the south, with the counter current to the north persisting on the continental break. During upwelling events, there is a flow in the first 30 m that is compensated at the deepest levels by a movement towards the coast [35–37]. The waters emerging on the continental shelf are from 120–150 m depth [35–37] and correspond to the subtropical branch of the Eastern North Atlantic Central Water, being colder, less saline and more nutrient enriched [26]. At an early stage, coastal upwelling develops as a thin band of cold waters along the coast [36]. Structures joining in stranded filaments (30 to 40 km wide and over 250 km long) and transported materials to the deep ocean begin to emerge with the persistence of N winds [38].

The morphology of the Portuguese continental margin has been studied by several researchers [39–43]. The ACS is formed by a flattened surface corresponding to a monoclinal with gentle slope to the West constituted by Cretaceous and Cenozoic formations. The monotony of the continental shelf is raised along the Furadouro by Cretaceous carbonate formations (Pontal da Galega and Pontal da Cartola), and in front of Mira by the Morraceiros relief, formed by Mesozoic and Cenozoic carbonate rocks (Fig 1) [40].

According to Dias et al. [44], the first reference to the sedimentary coverture of the continental shelf between Espinho and Cabo Mondego is found in the Lithological Submarine Chart published in 1914 (“Carta Litológica Submarina da Costa de Portugal de Leixões ao Cabo Mondego n° 2”). In the 1980s, several sedimentological studies [44–49] allowed to determine the unconsolidated sediments on the continental shelf between Nazaré and Minho River. Based on denser sampling, some works [50–53] enabled a more detailed identification and characterization of the sedimentary deposits in the study area. The sediment distribution and characteristics together with the knowledge on the processes that affect the depository of the PCS [49] were used to interpret the sedimentary dynamics of the region. These studies showed that sediments in the PCS between Espinho and Cabo Mondego are essentially sandy denoting energy levels that hinder the accumulation of fine sediments [50–56]. Indeed, the silt-clay fraction (<63 μm) generally increases from 80 m bathymetric to deeper areas that are hydrodynamically quieter environments [53]. Terrigenous particles predominate in the sandy fraction at the inner and mid continental shelf, where they reach percentages >90%, being quartz the most abundant component [49]. The biogenic component, formed essentially by mollusks and foraminiferal tests, becomes dominant below 100 m depth [49–53]. The authigenic component, represented by glauconite, occurs mainly on the outer continental shelf, the shelf break and on the upper continental slope [49–53]. Fine and medium sands occur along the Aveiro Canyon, on the continental shelf break and on the upper continental slope, while very fine sands predominate in the northern and southern sectors of the slope [49–51]. The coarse-grained particles (>2 mm), correlated with the paleo-mouth of the Vouga River and with paleo-coastlines, are arranged in two strips. The first one, roughly parallel to the present shoreline, is wider and well defined, located on the mid continental shelf (80–90 m), where gravel is sometimes the dominant textural class (> 50%). The second one is poorly defined, located on the outer continental shelf, where the percentage of gravel rarely exceed 25% [49–53]. The shallower gravelly strip is essentially composed by terrigenous particles consisting predominantly of quartz [49–53]. The gravel strip on the outer shelf is predominantly biogenic and comprises a relatively high amount of mollusks’ shells (fragmented, corroded and greyish) and foraminiferal tests (powered, greyish and sometimes glauconitized) [49–53].

Only a limited number of benthic foraminiferal studies has been carried out on the central western PCS (e.g. [54–57] The ACS has been previously studied by [53,58]. In particular, Martins et al. [58] analyzed the LAS in the ACS (Portugal; Fig 1).

## Material and methods

### Sampling, preparation and investigation

This study was developed under the Oceanographic Cooperation Agreement between Portugal and France (JNICT/ French Embassy, CNRS-INSU, CIRMAT), and the authorization of Administração do Porto de Aveiro (APA; Administration of the Aveiro Port) that gave the permission to collect bottom sediment samples from the NW Portuguese Continental Shelf off Aveiro (Portugal). No additional specific permissions were required for this work, which also does not involve endangered or protected species or vertebrates.

This study analyses data acquired in 44 sediment samples from NW Portuguese Continental Shelf off Aveiro (Portugal) at latitudes 40°30' N to 40°50' N and longitudes 8°46' W to 9°20' W, between 10 and 200 m depth (Fig 1). Samples were collected with a Reineck box corer, equipped with a stainless steel 172 x 85 mm box. Twenty-three sampling sites were located along 3 parallel transects (A1 to A3) in front of the Ria de Aveiro lagoon mouth from 10 to 51 m water deep (Fig 1; S1 Table). The 5 sites of transect B1 extends the depth distributions of the A-transects down to 95 m, while transects B2 (7 sites) and B3 (5 sites) range from 14 m to 190 m. The northernmost transect B4 (4 sites) cover a depth between 16 m and 68 m (Fig 1). A-transects were sampled 21 July and 2 August 1994 and B-transects between 28 July and 3 August, 1995, on board the oceanographic vessel “NO Côte d'Aquitaine” (CNRS—CIRMAT) of French nationality.

PVC tubes, 5 cm in diameter and 30 cm high, were pushed into the sediment collected at each station to sub-sample the first centimeter of surface sediments for granulometry, total organic matter content (TOM) and benthic foraminifera. For foraminiferal study, a volume of 10 cm^3^ of the first centimeter of surface sediment were immediately fixed with 4% formaldehyde in sea water (neutralized with sodium bicarbonate) and stained with rose Bengal (2 g/l) for staining living foraminifera [58].

Sediments for grain size analysis were dried in the oven at 50°C. The organic matter was removed from the sediments with hydrogen peroxide (H_2_O_2_) and carbonate minerals were dissolved using an ammonium acetate–acetic acid prior to grain-size analysis. For granulometric separation, about 250 g of dry sandy sediment and about 150 g of dry muddy sediment was weighted. The sediment fraction <63 μm was separated from the coarser one by wet sieving through a 63 μm mesh sieve. Both separated sedimentary fractions (>63 μm and <63 μm) were dried and weighted. Sedimentary fractions >63 μm were sieved using a column of sieves (125 μm, 250 μm, 500 μm, 1000 μm, 2000 μm) placed on a shaker for 15 min. The percentage of each fraction was determined based on the weight of each sediment fraction. Sediment mean grain size (SMGS) was evaluated according to [59].

The sediment for TOM analysis, stored at -20°C, was dehydrated in an oven at 100°C for about 24 hours [60]. The dry sediment was grinded in a mortar and homogenized. In calibrated crucibles (by exposure in a muffle at 450°C for 5 hours and cooled in a desiccator for 30 minutes), 1 g of the sediment of each sample was added. After weighing, the crucible and the sediment were placed in a muffle at 450°C for 5 h [60]. The crucibles and the sediment, taken from the muffle, cooled for 30 minutes in a desiccator, and weighed again. The weighing was carried out quickly to avoid significant variations due to humidity absorption. The TOM content was estimated by weight loss after the incineration and was expressed as percentage of dry weight of the sample.

For foraminiferal study, each sample was washed with water through sieves of 63 μm and 1000 μm to remove the formaldehyde, excess dye, fines and coarse sedimentary particles. Living and dead foraminifera, in the sediment fraction 63–1000 μm, were fully picked with a Pasteur pipette under a Zeiss binocular microscope, model Stemi SVII with a maximum magnification power of 264 times. The specimens were stored in foraminiferal microslides, identified and counted.

### Statistical methods

Species densities (standing crops) of LAs and DAs are based on 10 cm^3^ sediment. Similarities in composition between LAs and DAs were measured for sample *j* using the Cosine [61] between species *i*, because being an angular measure it becomes independent from differing sample densities that are represented as vector lengths.

$${cos}_{living\;dead,j}=\sum_{i=1}^{k}\left(n_{i\;living}/\sum_{i=1}^{k}n_{i\;living}^{2})∙(n_{i\;dead}/\sum_{i=1}^{k}n_{i\;dead}^{2}\right)$$ (1)

Differences in ranking of species between both assemblages in sample *j* were determined by Spearman’s rank distances *d* averaged by species number *k* and tested for significance using Spearman’s Rank Correlation Coefficient [62].

$$d_{j}^{2}=1/k_{j}∙\sum_{i=1}^{k_{j}}{\left({rank}_{i\;living}-{rank}_{i\;dead}\right)}^{2}$$ (2)

Accepting the *H*_0_-hypothesis of non-correlation confirms the grade of disorder in rankings between LAs and DAs. Moreover, all negative correlations corroborate the disorder in rankings, particularly when negative correlations are significant [21].

While the Cosine measures similarities in accordance (${cos}_{j}^{2}=\%\;{accordance}_{j}$), the rank distance as a correlation measured with negative values shows the intensity of disorder in rankings, thus representing a measure for differences between LAs and DAs.

Alpha diversities [63–64] of LAs and DAs at a sampling site were calculated using the Chao1-Index [65]

$$S_{Chao\;1}=S_{observed}+f_{1}(f_{1}-1)/2(f_{2}+1)$$ (3)

for measuring species richness (*S)*, where *f*_1_ characterizes the number of species represented by a single specimen and *f*_2_ the number of species with two specimens. For heterogeneities, the Evenness measure developed by Buzas and Gibson [66] based on the Shannon Entropy (*H*) was used

$$H^{\prime }=e^{H}/S_{observed}$$ (4)

Combinations of indices for densities, similarities and diversities enlighten the relationships between LAs and DAs [21]. Diversity diagrams show species richness for sample *j* standardized over the complete data set (all LAs and DAs under investigation) on the *x*-axis

$$x_{j\;living,dead}^{*}=(x_{j\;living,dead}-{\bar{x}}_{total})/\sigma_{x\;total}$$ (5)

and the corresponding standardized heterogeneities on the *y*-axis

$$y_{j\;living,dead}^{*}=(y_{j\;living,dead}-{\bar{y}}_{total})/\sigma_{y\;total}$$ (6)

In the above-mentioned coordinate system starting from the LAs, the vector between both standardized diversity measures (species richness and heterogeneity) demonstrates intensities of relations between LAs and DAs. It is determined for sample *j* by

$${Length}_{j\;living\;dead}=\sqrt{{\left(x_{j\;living}^{*}-x_{j\;dead}^{*}\right)}^{2}+{\left(y_{j\;living}^{*}-y_{j\;dead}^{*}\right)}^{2}}$$ (7)

and

$${Angle}_{j\;living\;dead}=arctan[\left(y_{j\;living}^{*}-y_{j\;dead}^{*})/(x_{j\;living}^{*}-x_{kj\;dead}^{*}\right)]$$ (8)

measured in radians [21].

The grade of integration of living individuals into the DAs is measured by the ‘Incorporation Value’, which weighs the proportion of living individuals and total assemblages by similarities between both assemblages, characterizing the *j*^*th*^ sample by

$${IncorpVal}_{j}={Similarity}_{j\;living\;dead}\cdot {density}_{j\;living}/\left({density}_{j\;dead}+{density}_{j\;living}\right)∙200$$ (9)

where every similarity index varying between 0 (completely dissimilar) and 1 (completely similar or identical) can be used. This value normally ranges between 0 and 100, but can exceed the upper limit when the density of living individuals is higher than of dead individuals.

Second, similarities in composition can be related to the lengths of the standardized vectors by

$${SimDivers}_{j\;living\;dead}={Similarity}_{j\;living\;dead}/({Length}_{j\;living\;dead}+0.01)∙100$$ (10)

according to [21].

Comparing sites or groups of sites by diversities is named beta diversity [63]. Several approaches have been developed, the one concentrating on presence/absence data (incidence based), the other (abundance based) include frequencies [67]. Along transects, beta diversities can be calculated between pairs of succeeding sample sites. We calculated one index using incidence-based data. Species turnover between samples following Tuomisto [68] can be reduced in pairwise comparisons to

$$\beta_{T}=(S_{1}-c)+(S_{2}-c)$$ (11)

with *S*_1_ = number of species in sample 1, *S*_2_ = number of species in sample 2 and *c* = number of common species.

Using abundance-based data, we calculated the Hill numbers ^0^*D* and ^1^*D* for diversities derived from the general diversity measure

$${}^{q}D={\left(\sum_{i=1}^{S}p_{i}^{2}\right)}^{1/\left(1-q\right)}$$ (12)

introduced by Hill [69]. The parameter *D* determines the number of dominant species decreasing with increasing constant *q*. When *q* = 0, *D* represents the total number of species in samples because of equal weighting. With increasing *q*, the number of dominant species decreases leading to a similarity profile with *q*-values at the abscissa [67]. According to Jost et al. [67] the limit of Eq 12 for *q* = 1 is the exponent of the Shannon entropy (*H*).

Beta diversity [70] comparing successive sample sites is defined by

$${}^{q}D_{\beta }={}^{q}D_{\gamma }{/}^{q}D_{\alpha }$$ (13)

As the Hill number ^0^*D* represents the species number in alpha-diversities, ^0^*D* equals in pairwise comparison (beta-diversities) the species turnover of Eq 12. Because ^1^*D* equals the exponent of the Shannon entropy [67]. Beta diversity reduces to exp *H*_*γ*_ −exp *H*_*α*_.

As all sample sites are located along transects, frequency histograms based on unequal depth intervals (0–20 m, 20–40 m, 40–60 m, 60–100 m, 100–150 m and 150–200 m) were calculated for the most abundant species (>100 specimens). Disparity of intervals is caused by the intense scatter of sites in shallower regions and rareness of sites in deeper regions (Fig 1). Interval averages were used to estimate mean frequencies of species for the whole transect. Based on mean densities in the above intervals, a set of *k* normal distributions

$${f(x)}_{i}=\sum_{k=1}^{k=m}d_{ik}∙exp(-\frac{{\bar{x}}_{ik}^{2}}{2s_{ik}^{2}})$$ (14)

where *i* indicates the species and *x* the depth, were used to fit the empirical distributions. Distribution ranges of living and dead foraminifera within a species could be calculated using the lower and upper 0.5 and 99.5 percentiles of the complete set of normal distributions. Mean density $\bar{d}$ of the *i*^*th*^ species weighted by the standard deviation was calculated using:

$${\bar{d}}_{i}=\sum_{k=1}^{k=m}\left[\left(d_{ik}^{2}s_{ik}\right)/\sum_{k=1}^{k=m}d_{ik}s_{ik}\right]$$ (15)

Percentages of living foraminifera on dead specimens were calculated based on the theoretical frequency distributions in the interval between 0 m and 200 m.

## Results

### Depth, grain size and total organic matter

**Fig 2:**
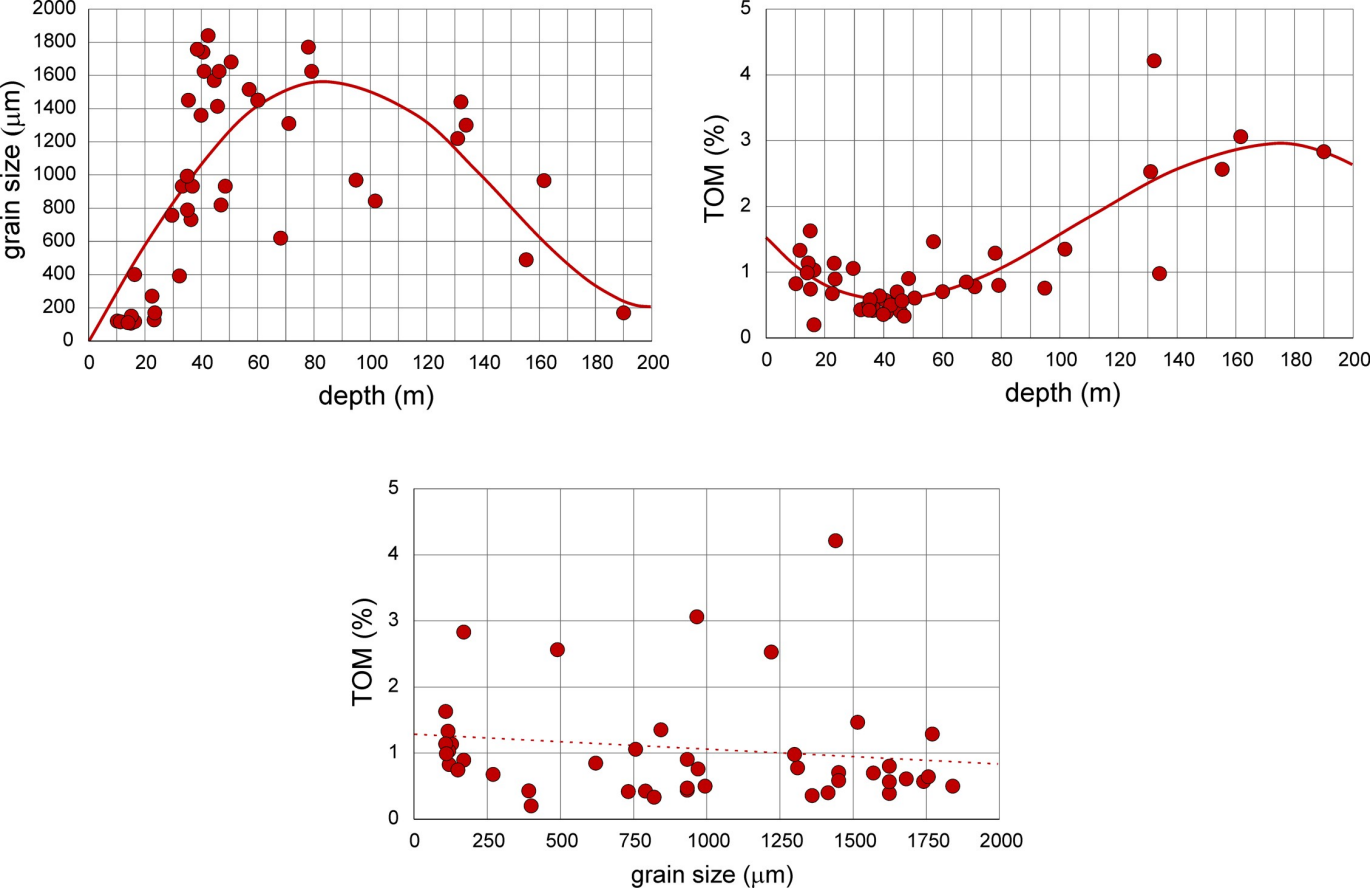
Correlations between depth, sediment mean grain size (reported as grain size) and total organic matter (TOM). Full line: significant correlation; broken line: non-significant correlation.

The environmental factors (S1 Table), namely water depth (10–190 m), SMGS (108–1840 μm) and TOM (0.20–4.21%) were inter-correlated to evidence their influence on LAs and DAs (S2 Table) in the dependence of each other (Fig 2; S3 Table). The SMGS correlates significantly [*p*(*H*_0_) = 7.82E-10] with water depth using the 4^th^ order power function (Fig 2). Starting from 10 m to 30 m with ‘fine and medium sand’, SMGS tops with ‘very coarse sand’ between 40 m and 80 m, then decreases to ‘medium and fine sand’ from 140 m to the deepest sites of the area (Fig 2). Percentages of TOM also correlate significantly [*p*(*H*_0_) = 3.13E-13] with water depth using a 3^rd^ order power function (Fig 2). Starting with 1.5% in nearshore stations, minimum values (~0.5%) of TOM are found between 30 m and 60 m. Percentages of TOM increase with depth and reach maximum values of ~3% between 150 m and 200 m (Fig 2). Percentages of TOM do not correlate with SMGS (Fig 2) neither in linear regression [*p*(*H*_0_) = 0.238] nor in 6^th^ order power regression [*p*(*H*_0_) = 0.054]. Due to the non-normal frequency distribution, relations to grain size can be characterized by a median of 0.79% supported by the 1^st^ quartile of 0.5% and the 3^rd^ quartile of 1.3% TOM.

### Densities

**Fig 3:**
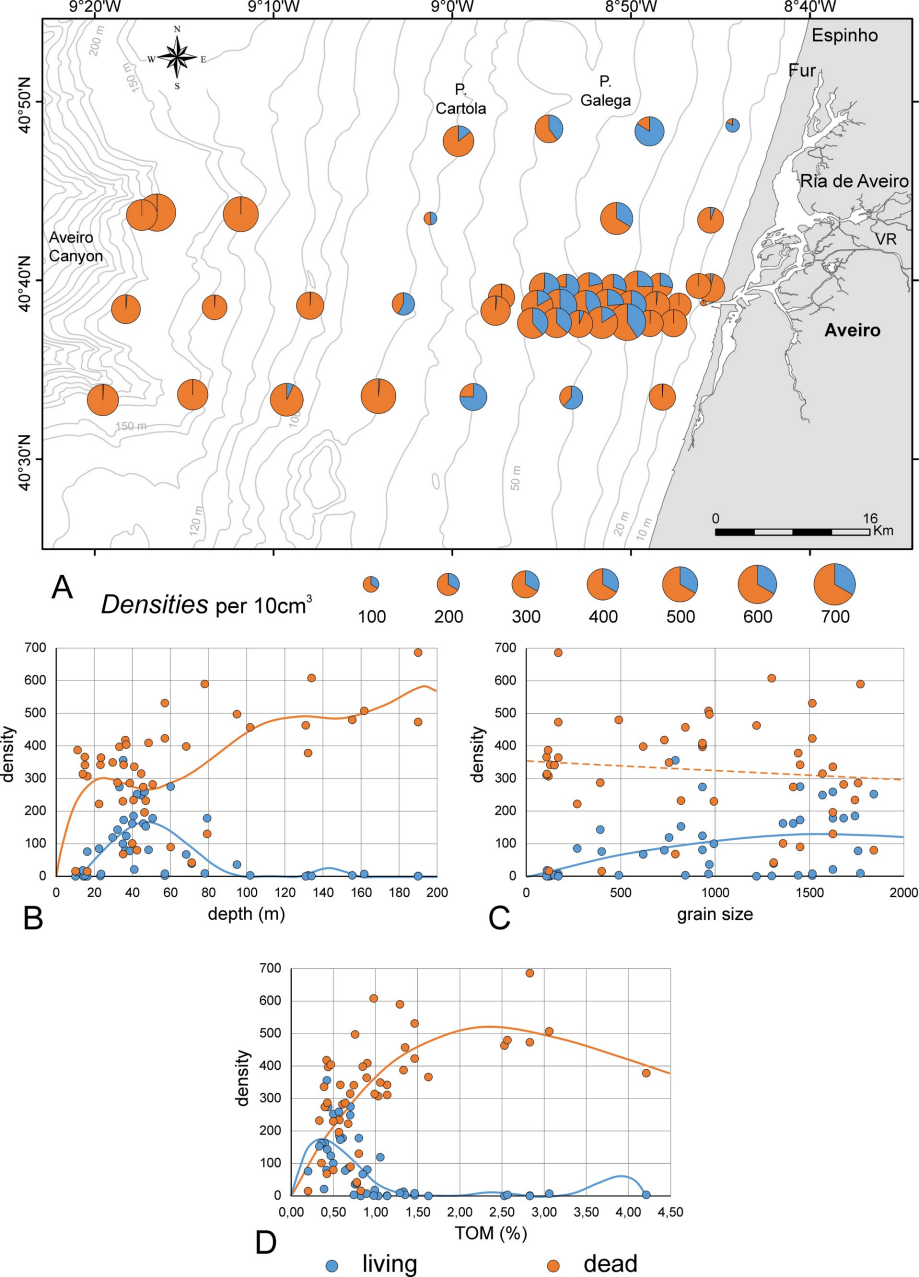
Densities of assemblages in the investigated area. A. Densities of living and dead individuals represented as pie charts. B. Dependence of densities from depth fitted by 6^th^ order power functions. C. Dependence of densities from grain size fitted by 2^nd^ order power functions (living) and linear regression. D. Dependence of densities from TOM fitted by 6^th^ order (living) and 3^rd^ order (dead) power functions. Full line: significant correlation; broken line: non-significant correlation.

A total of 3,984 living and 14,979 dead individuals are divided into 236 species (S2 Table). Only 35 species exceed the representative numbers of 100 specimens, whereof 16 species are represented by >200 individuals. Ninety-nine species are represented by <5 specimens and 45 ones by a single specimen. This explains the high gamma diversity [63], [71], in species richness (Chao1_combined_ = 290.5 species) combined with low heterogeneity (Evenness _combined_ = 0.195, Evenness _living_ = 0.206, Evenness _dead_ = 0.211). Densities of living individuals are remarkably high for sites of transects B2 and B4 between 30 m and 50 m, which is supported by high proportions at these depths in all A-transects (Fig 3A, S3 Table).

Depth dependence of densities are supported by non-linear regression, where 6^th^ order power functions significantly fit the trends in LAs [*p*(*H*_0_) = 3.46E-05] and DAs [*p*(*H*_0_) = 3.13E-07]. Densities of living individuals are low at 10 m (~15 specimens) and get the averaged maximum of ~160 specimens at 45 m. The following decreasing trend to 100 m is gradual but then drops to extremely low mean densities (~2 specimens) until 200 m (Fig 3B).

A different trend is identified on dead individuals, where after a rapid increase to 20 m (~300 individuals), densities slowly decrease to mean densities of 280 specimens at 50 m, then continuously increase to 540 specimens at 190 m water depth (Fig 3B).

Only in LAs, dependence of densities from SMGS are weakly significant [*p*(*H*_0_) = 1.69E-03] as proven by 2^nd^ order power regression (Fig 3C) getting a mean maximum with 129 individuals at 1651 μm grain size (2^nd^ order power function). Densities of DAs varying around a median of 341.5 specimens show no relation to SMGS and are bordered by the 1^st^ quartile (231.5 specimens) and 3^rd^ quartile (419.25 specimens) along the gradient (Fig 3C). Percentages of TOM significantly influence densities in both assemblages (Fig 3D) following 6^th^ order (living, *p*(*H*_0_) = 4.05E-08) and 3^rd^ order (dead, *p*(*H*_0_) = 8.22E-06) power regressions. Dead individual number tops with a mean of ~520 specimens at 2.5% of TOM, while in living individuals the regression function increases reaching a mean maximum of ~170 individuals at 0.4% of TOM, then it decreases rapidly (Fig 3D).

### Similarity

**Fig 4:**
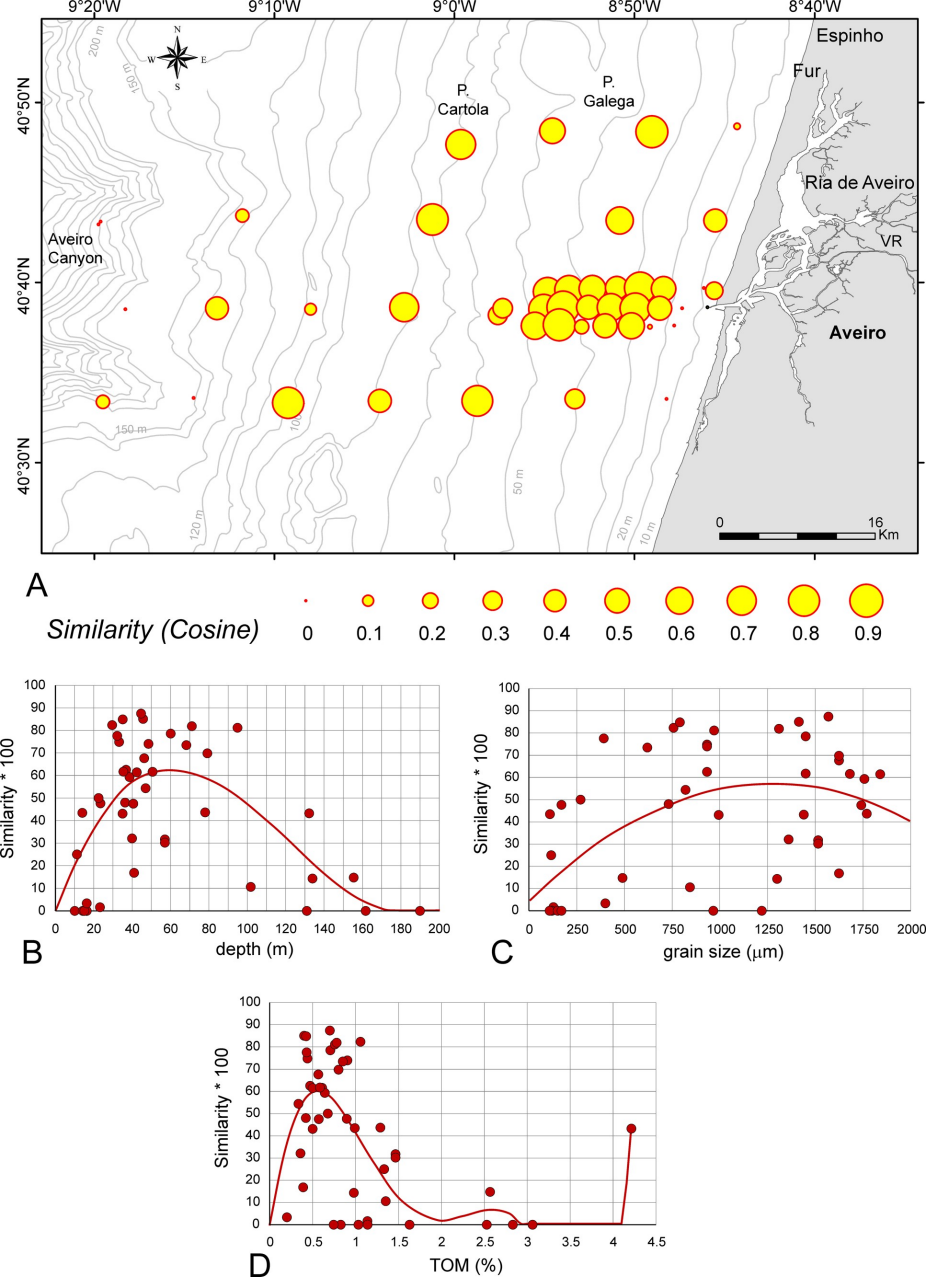
Similarities between assemblages’ density in the investigation area. A. Similarities represented as circle areas. B. Dependence of similarities from depth fitted by 3^rd^ order power function. C. Dependence of similarities from grain size fitted by 2^nd^ order power function. D. Dependence of similarities from TOM fitted by 6^th^ order power function. Full line: significant correlation.

Similarities between the density of LAs and DAs in the investigation area are presented in Fig 4A. Depth dependence of similarities between LAs and DAs can be significantly [*p*(*H*_0_) = 5.28E-06] fitted by a 3^rd^ order power function (Fig 4B). A strong increase of similarity between 0 and 40 m peaks with a mean maximum of ~60 individuals between 50 and 70 m, then slowly decreasing to zero similarity at the deepest site, because living individuals are absent (S3 Table).

The dependence of similarities from grain size is less significant [*p*(*H*_0_) = 1.37E-05] being fitted by a 2^nd^ order power function, getting its mean maximum (cosine = 0.58) at 1250 μm (Fig 4C).

Similar to the dependence of TOM from depth, its dependence from grain size can also be significantly [*p*(*H*_0_) = 9.28E-07] fitted by a 6^th^ order power function (Fig 4D). A strong increase of similarity between 0.0 and 0.4% of TOM is found with peaks at 0.6% TOM. A decrease of similarity at 1.6% TOM is followed by no individuals at deeper sites, with the exception of 15 specimens at a site characterized by 15% TOM and the exception of 44 individuals at 4.2% TOM (Fig 4D).

### Rank distance

**Fig 5:**
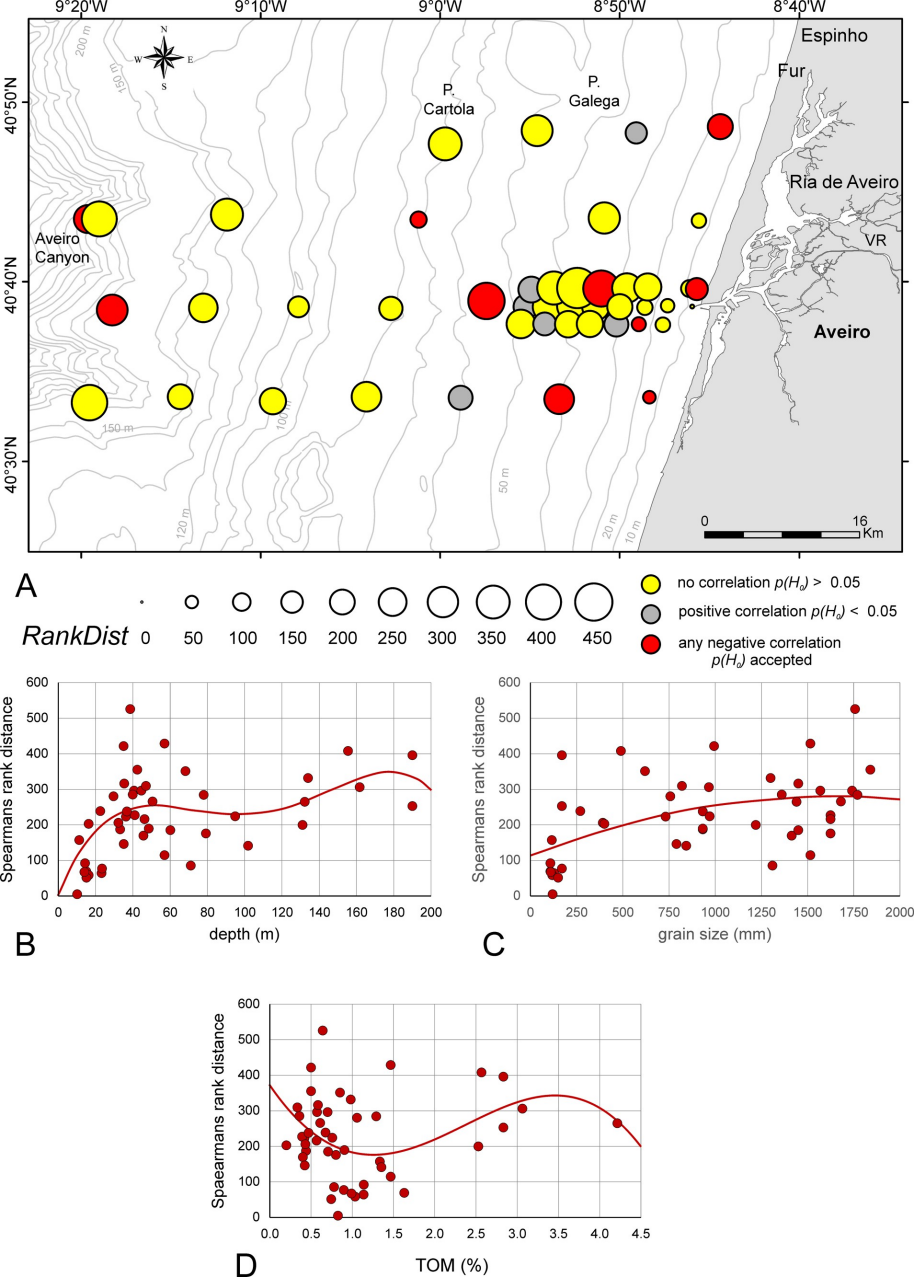
Rank distances between assemblages in the investigation area. A. Rank distances represented as circle areas. B. Dependence of rank distances from depth fitted by 4^th^ order power function. C. Dependence of rank distances from grain size fitted by 2^nd^ order power function. D. Dependence of rank distances from TOM fitted by 3^rd^ order power function. Full line: significant correlation.

Rank distances between assemblages in the investigation area are presented in Fig 5A, as well as the dependence of rank distances from depth (Fig 5B), SMGS (Fig 5C) and TOM (Fig 5D). The disorder in rankings in dependence of depth is significant [p(H_0_) = 1.25E-05] because described by a 5^th^ order power function (Fig 5B). After a strong increase, the first local maximum (rank distance ~250) is obtained at 50 m. Towards deeper sites, the rank distances slowly increase reaching its maximum (350) at the deepest sites. The dependence of rank distances from grain size is less significant [*p*(*H*_0_) = 8.21E- 04] using 2^nd^ order power functions (Fig 5C). Starting with a mean rank distance of 150 at 100 μm grain size, the function peaks with a mean rank distance of 280 at ~1600 μm grain size. The dependence of rank distances from TOM is significant [*p*(*H*_0_) = 0.016] when fitted by a 3^rd^ order power function (Fig 5D). Starting with rank distances of approximately 300 around 0.5% TOM, a local minimum with rank distance of 180 is identified between 1.0% and 1.5% TOM. After an increase, a local maximum of rank distance (345) occurs around 3.5% TOM.

### Incorporation values

**Fig 6:**
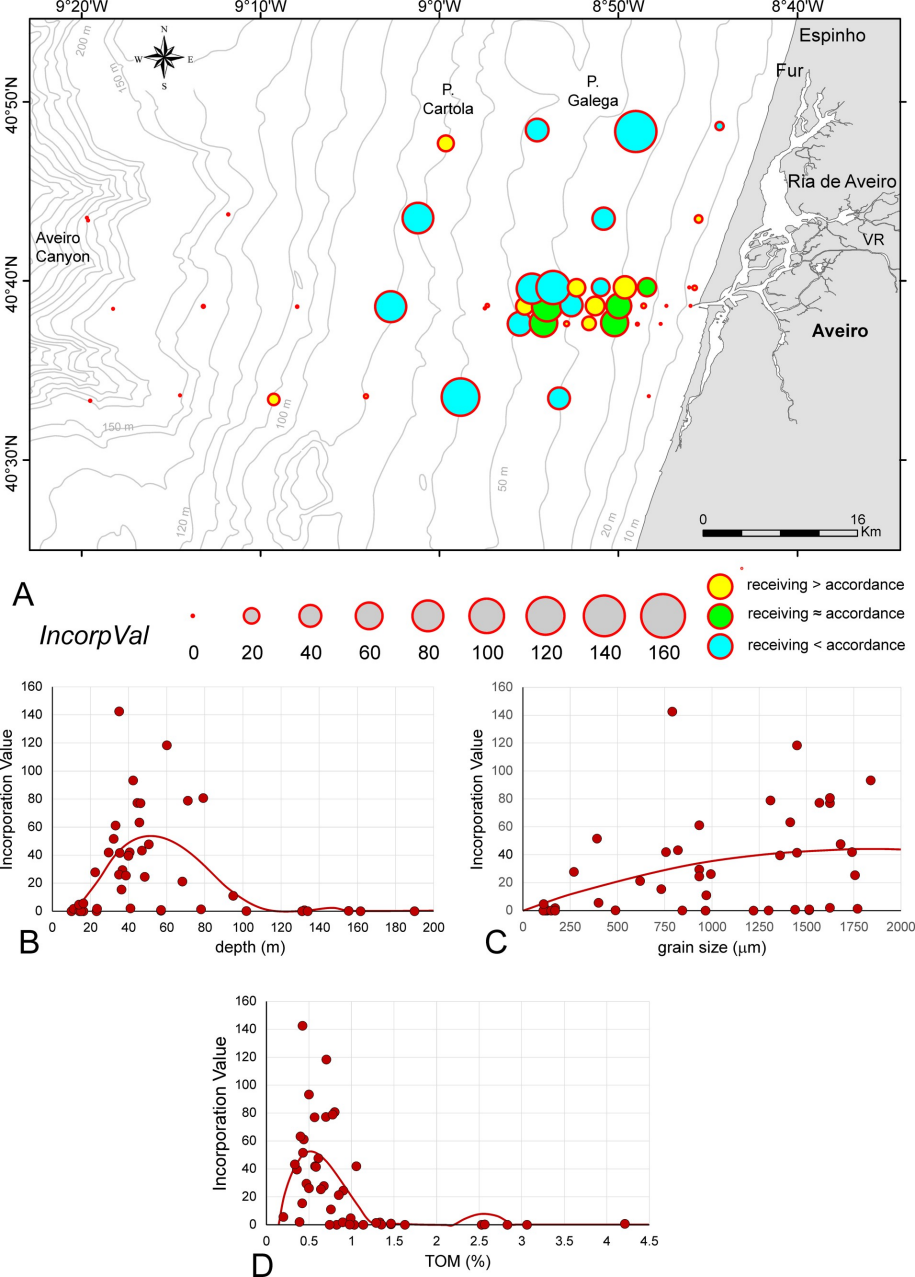
‘Incorporation Values’ of sites in the investigation area. A. ‘Incorporation Values’ represented as circle areas. B. Dependence of ‘Incorporation Values’ from depth fitted by 6^th^ order power function. C. Dependence of ‘Incorporation Values’ from grain size fitted by 2^nd^ order power function. D. Dependence of ‘Incorporation Values’ from TOM fitted by 5^th^ order power function. Full line: significant correlation.

The IncorpVals weighting the proportion of living on total specimens by similarities are mapped in Fig 6A (S3 Table). Dependence of IncorpVals from depth can be significantly [*p*(*H*_0_) = 1.04E-05] fitted by 6^th^ order power functions (Fig 6B) starting with zero values at 10 m and reaching a mean maximum (55) at 50 m depth. Zero values are again observed at 100 m and continue until 200 m, interrupted by an extremely weak second maximum (2) at 135 m. IncorpVals significantly correlate with grain size [*p*(*H*_0_) = 2.60E-03] using a 2^nd^ order power regression (Fig 6C). Starting with low values at 100 μm grain size, the mean optimum (IncorpVal = 45) is attained at 1850 μm grain size. Moreover, sites with IncorpVal = 0 are spread over the whole grain size scale in the investigation area (Fig 6C). The dependence of IncorpVals from percentages of TOM is peculiar, which can be significantly [*p*(*H*_0_) = 4.95E-06] fitted by a 5^th^ order power function (Fig 6D). This function starts at 0.2% TOM, reaches the maximum (52) at 0.5% TOM and falls down to 0 at 1.5% or higher TOM. The small peak in the power function at 2.5% TOM is artificial caused by function properties.

### Similarity/Diversity index

**Fig 7:**
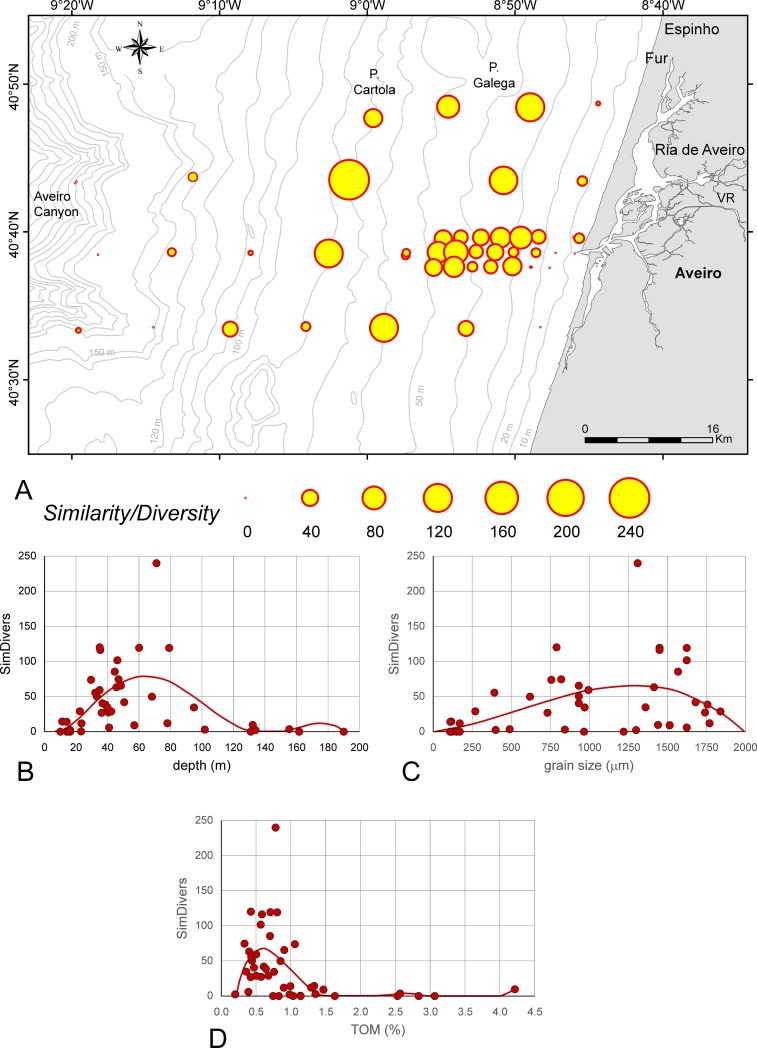
Similarity/Diversity index (SimDivers) of sites in the investigation area. A. SimDivers represented as circle areas. B. Dependence of SimDivers from depth fitted by 5^th^ order power function. C. Dependence of SimDivers from grain size fitted by 3^rd^ order power function. D. Dependence of SimDivers from TOM fitted by 6^th^ order power function; full line: significant correlation.

Weighting of similarities by diversities (SimDivers), following Eq 9, are mapped in Fig 7A (S3 Table). The dependence of SimDivers’ from depth can be significantly [*p*(*H*_0_) = 2.29E-05] fitted by a 5^th^ order power function (Fig 7B). Starting at 10 m, the function maximum (SimDivers = 75) is located at 65 m. The decrease of the function to 0 at 140 m is not documented in the observed values, where 0 values start from 160 m downwards because living specimens lack at these depths. A significant [*p*(*H*_0_) = 1.80E-04] power function of the 3^rd^ order describes the relation between the SimDivers Index and grain size. The function maximum (SimDivers = 65) occurs at 1350 μm, then decreasing to 0 at 2000 μm (Fig 7C). The relation of SimDivers to TOM can be significantly [*p*(*H*_0_) = 1.80E-04] fitted by a 6^th^ order power function (Fig 7D). Starting at 0.2% TOM, the main function maximum (SimDivers = 65) is reached at 0.55% TOM. This function decreases to 0 at 1.5% TOM. Two local maxima around 2.5% (SimDivers = 4) and 4.2% TOM (SimDivers = 10) interrupt the 0 values of SimDivers.

### Diversity diagrams

**Fig 8:**
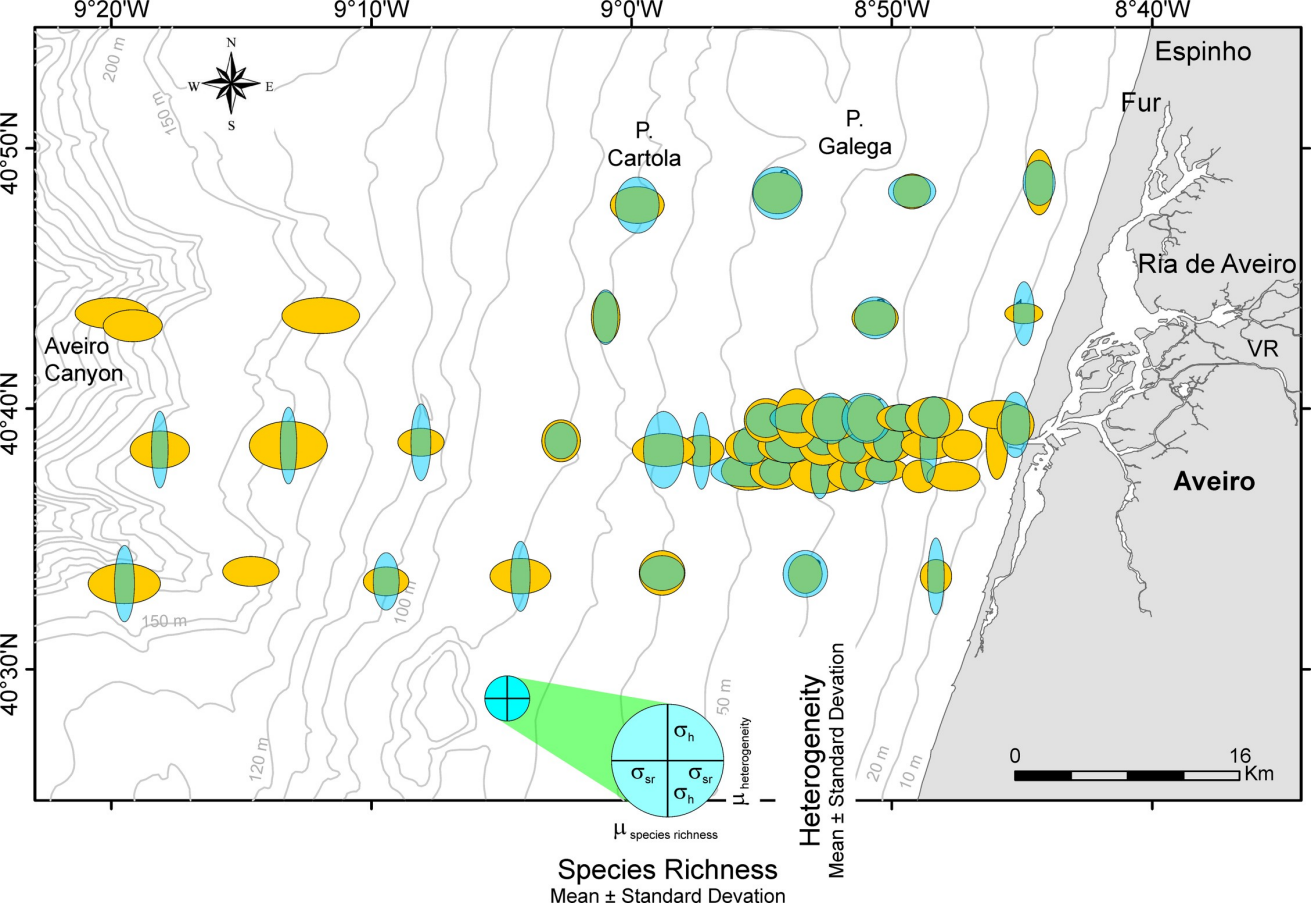
Diversity diagrams of sample sites in the investigation area based on standardized species richness’ and heterogeneities. Living assemblages in light blue, dead assemblages in orange. Species richness based on Chao1 [65] with $\bar{x}$ = 43.84 and *σ* = 22.52; heterogeneity based on Evenness [66] with $\bar{x}$ = 0.573 and *σ* = 0.203. The blue circle indicates standardized means ($\bar{x}$ = 0) and standard deviations (*σ* = 1) for both species richness (abscissa) and heterogeneity (ordinate).

Species richness and heterogeneity of LAs and DAs standardized over the total number of LAs and DAs are represented in diversity diagrams, where species richness is denoted on the *x*-axis and heterogeneity on the *y*-axis (Fig 8, S3 Table).

**Fig 9:**
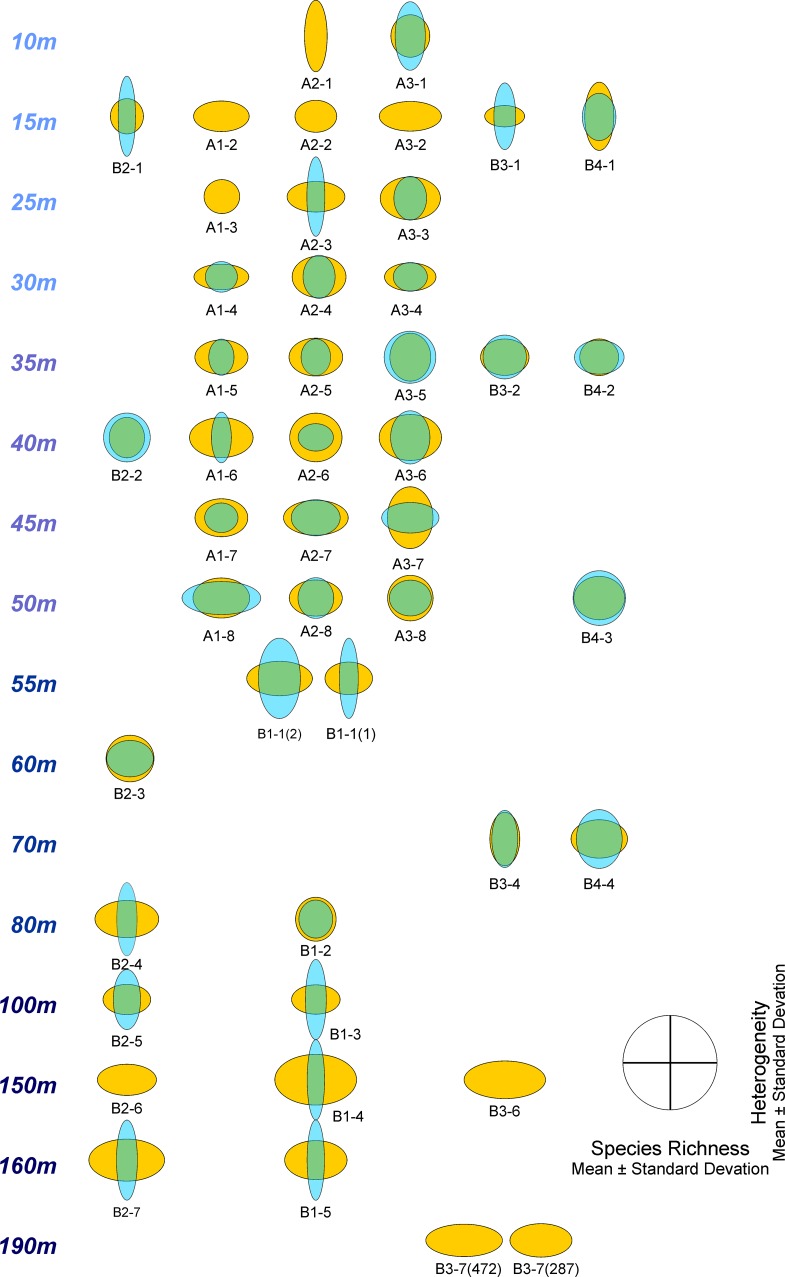
Diversity diagrams of sample sites in Fig 8 arranged by transects and water depth. Living assemblages in light blue and dead assemblages in orange.

General trends in the relations between LAs and DAs can be better described using Fig 9, where the transect diagrams are separated by depth and transect. In the shallowest sites (~10 m) of A-transects, species richness is low combined with high heterogeneity in DAs, while LAs are missing or having the same diversities as DAs. Diversity diagrams of sites in the B-transects show similar configurations at 15 m comparable to sites in the A-transects at 10 m, while diagrams in the A-transects at 15 m are devoid of living individuals and high species richness combined with low heterogeneity in DAs (Fig 9). From 25 m to 50 m species richness (high) and heterogeneities (low) remain rather constant in DAs within the A-transects. The LAs behave dissimilarly starting with low species richness and high heterogeneities from 25 to 35 m, becoming more similar to DAs from 40 to 50 m. The coincidence of diversity diagrams in LAs and DAs is remarkable for sites in all B-transects between 35 and 60 m. Sites A3-5 at 35 m and A3-8 at 50 m belong to this group. This trend continues in sites B1-2, B2-3 and B4-4 from 60 m to 80 m (Fig 9). Diversity diagrams show an increase of species richness combined with constant low heterogeneities in DAs toward deeper sites (70 m to 190 m), while species richness decreases in LAs to 0 combined with an increase in heterogeneities (Fig 9).

Relations between LAs and DAs expressed in diversity diagrams and their dependence on environmental factors are represented in a coordinate system spanned by the two most important coordinates obtained by nonmetric multidimensional scaling (nMDS). Scaling is based on Euclidean Distances, because the variables species richness and heterogeneity are standardized over all LAs and DAs.

**Fig 10:**
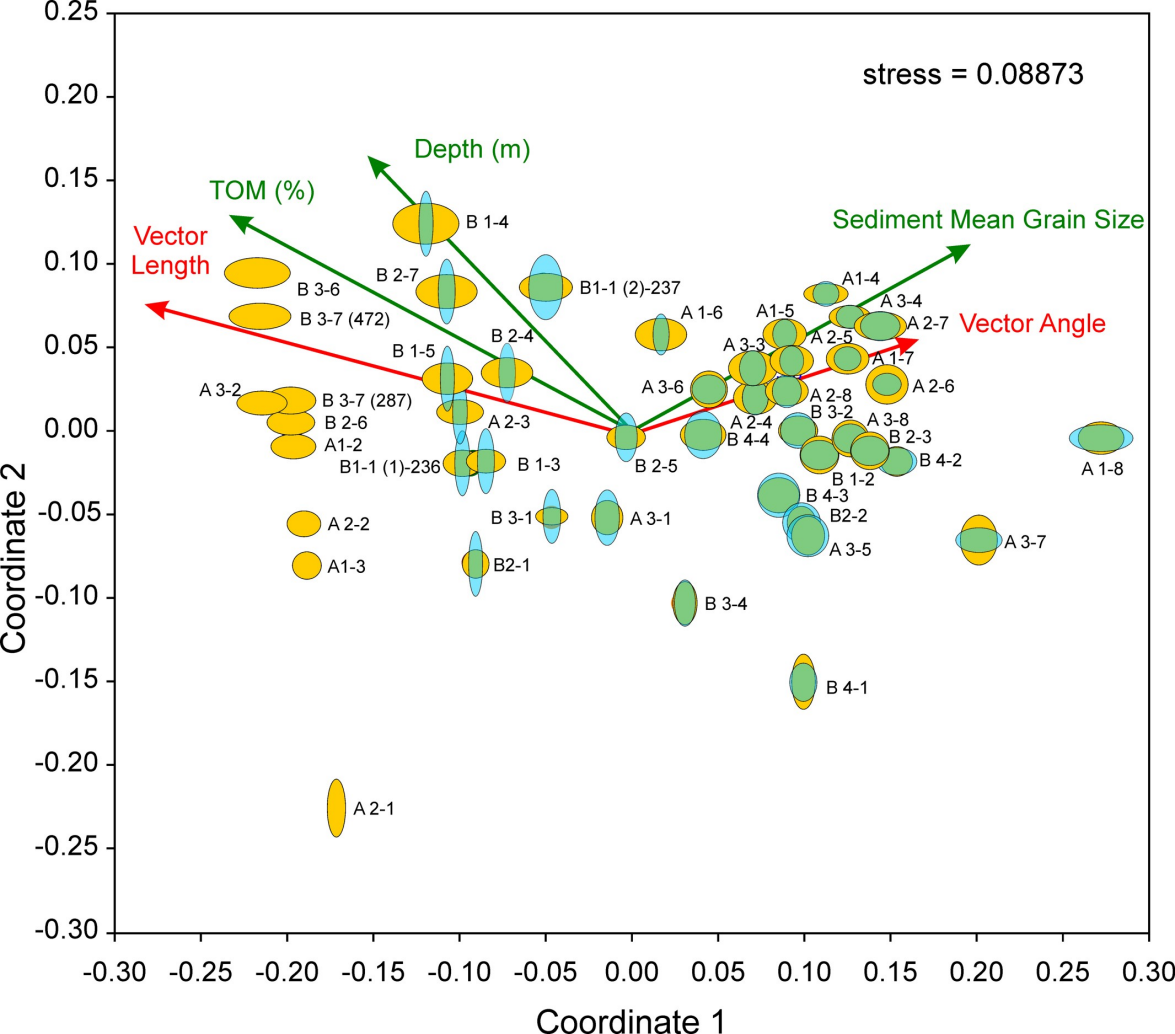
Diversity diagrams of sample sites positioned in a coordinate system obtained by nMDS. Influence of environmental factors water depth, SMGS and TOM represented as vectors together with the variables standardized diversity vector’s length and angle. Living assemblages in light blue and dead assemblages in orange.

The resulting diagram (Fig 10) perfectly represents the reduction of the original 4-dimensional space (*species richness*_*living*_, *species richness*_*dead*_, *heterogeneity*_*living*_, *heterogeneity*_*dead*_) into 2 dimensions supported by a stress of 0.089.

The strongly correlated environmental factors water depth and percentages of TOM are mainly represented along coordinate 1. Here, the trend in LAs and DAs from almost coincident diversities in LAs and DAs at high coordinate values to high species richness/low heterogeneities in DAs and low to missing species richness coupled with high heterogeneities in LAs at low coordinate is characteristic. Following the grain size vector that is almost independent from depth and TOM, the increase of species richness with more or less constant heterogeneities is characteristic for DAs. Along this gradient, LAs start with species numbers of 0 at low gradient values becoming almost identical with the high species numbers of DAs at highest gradient values. On the contrary, heterogeneities of LAs are high at low values of the grain size vector and approximate heterogeneities of DAs at high grain size values (Fig 10).

### Beta diversities

**Fig 11:**
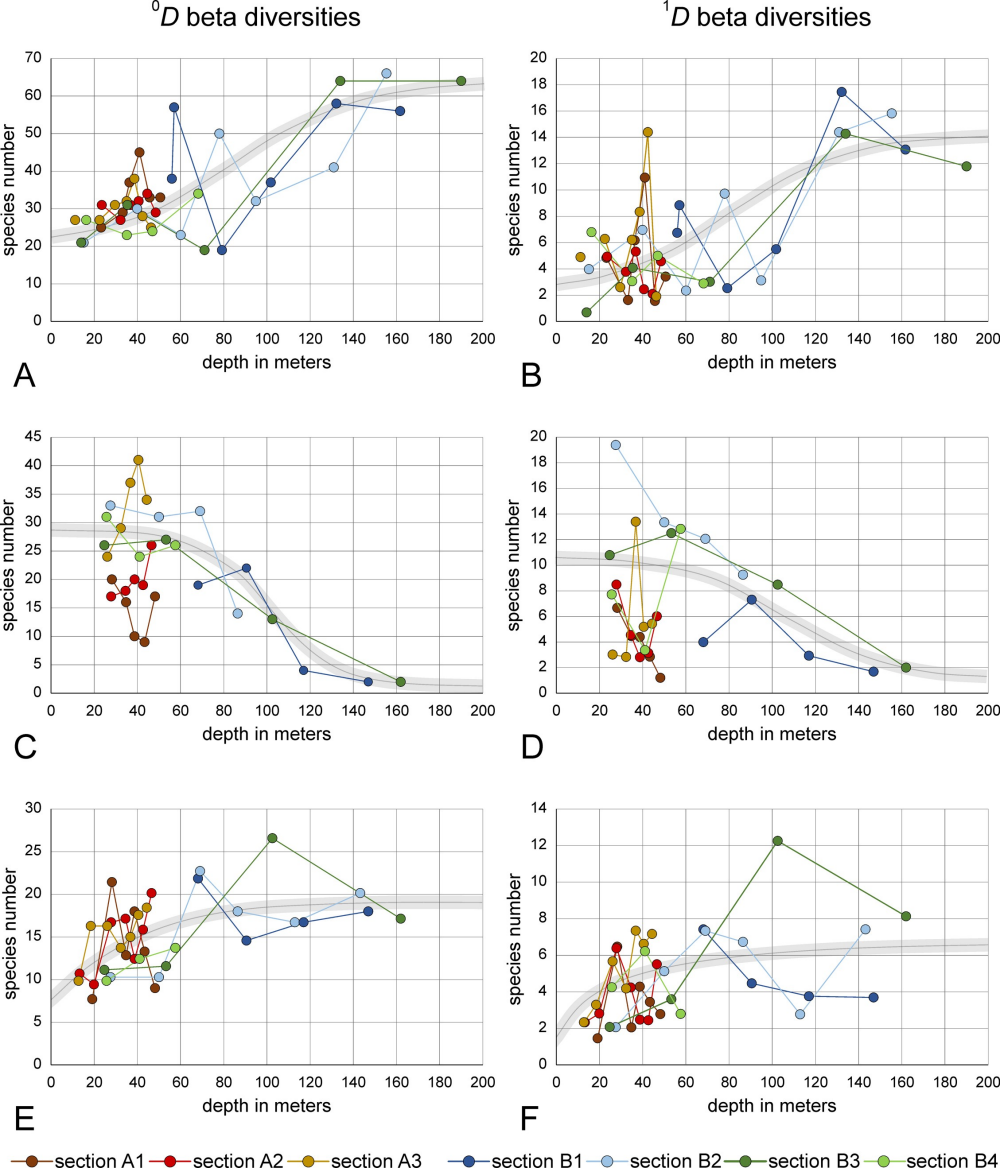
Beta diversities along transects determined by Hill numbers ^0^*D* (species number) and ^1^*D* (dominant species). A, B. Comparison between LAs and DAs of transect sites. C, D. Comparison between LAs of succeeding sites along transects. E, F. comparison between DAs of succeeding sites along transects. Grey lines: fit by logistic functions.

Beta diversities between assemblages define the spatial change along transects. The Hill numbers ^0^*D* and ^1^*D* were used for pairwise comparisons of succeeding sites (S4 Table). While ^0^*D* expresses the turnover rate in species number, ^1^*D* defines the turnover in number of dominant species that is based on Shannon’s Entropy [67]. All increases and decreases in turnover values could be modelled by logistic functions (Fig 11).

On the basis of the comparison of beta diversities between LAs and DAs of single sites (Fig 11A and 11B), species turnover is generally in favor of DAs (S4 Table). The turnover in species number (^0^*D*) starts at the surface with a mean of 20.2 species coming up to a mean of 64.3 species at 200 m (Fig 11A). Fitting by logistic functions is significant with *p*(*H*_0_) = 1.58E-06. The turnover in dominant species (^1^*D*) can be significantly [*p*(*H*_0_) = 3.79E-06] fitted by a function similar to the species turnover ^0^*D*, but characterized by a lower mean (2.2 species) near the surface that increases to 14.3 species at 200 m (Fig 11B). The negative correlation between turnover in species number and the similarity between LAs and DAs is significant [*p*(*H*_0_) = 0.0040].

Regarding LAs, beta diversities of successive transect sites show higher species turnover in shallow regions, followed by a decrease to a few species at the deepest transect sites (Fig 11C and 11D). Species turnover ^0^*D* starts with a mean of 28.6 species in shallowest regions keeping the turnover rate rather constant until 60 m. A rapid decrease between 60 from 26.8 species at 60 m to 4.8 species at 130 m is followed by slowly decreasing species numbers culminating with 1.2 species at 200 m (Fig 11C). Fitting this trend by logistic function is significant with *p*(*H*_0_) = 7.24E-05. The turnover ^1^*D* in dominant species can be fitted by a logistic function similar to the turnover ^0^*D*, but being not significant with *p*(*H*_0_) = 0.135. Species numbers start with a mean of 10.6 in the shallowest region, strongly decrease between 50 and 140 m and ends with 1.4 dominant species at 200 m (Fig 11D).

The turnover trend in DAs is opposite to LAs caused by the increasing number of dead individuals towards deeper sites (Fig 11E and 11F). Species turnover ^0^*D* starts with a mean of 8.9 species in shallowest regions steadily increasing to 22.0 species at 140 m approximating constancy in deeper parts (22.2 species at 200 m). Fitting this trend by logistic function is significant with *p*(*H*_0_) = 5.93E-04 (Fig 11E). A similar trend can be found for the turnover rate ^1^*D* for dominant species. The rapid increase starting with a mean of 1.4 dominant species at the surface to 5.5 species at 60 m is followed by a continuously weakening increase rate, ending with 6.5 species at 200 m (Fig 11F). Again, the fit by a logistic function is significant with *p*(*H*_0_) = 0.004.

### Species abundance

**Fig 12:**
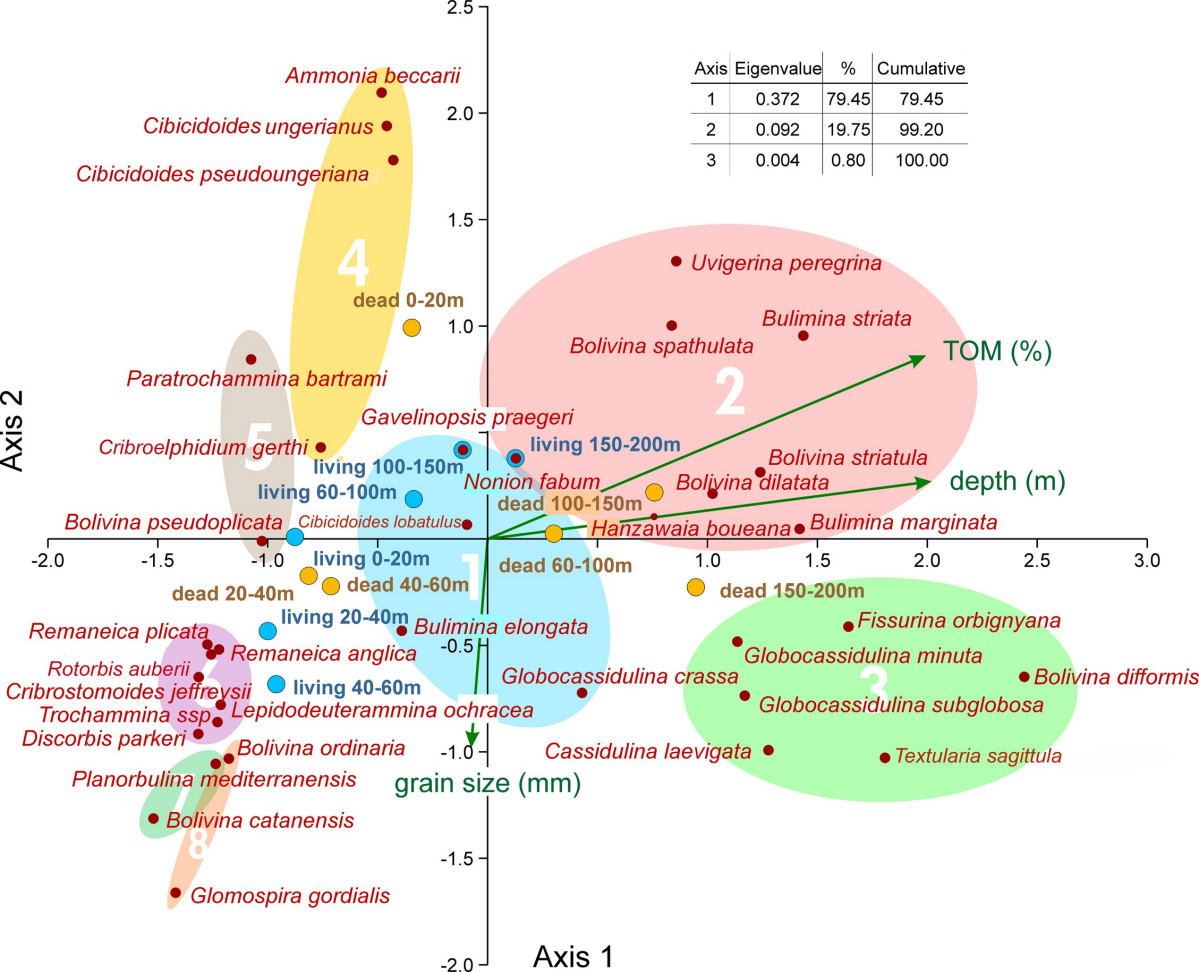
Canonical correspondence analysis of the most abundant species based on depth distributions of living and dead individuals. Groups are obtained by hierarchical cluster analysis Unweighted Pair Group Method with Arithmetic Mean using Cosine measures.

The mean density of living and dead individuals was calculated for the 35 most abundant species in the depth intervals 0–20 m, 20–40 m, 40–60 m, 60–100 m, 100–150 m and 150–200 m. Frequency distributions of species were compared using Canonical Correspondence Analysis including the environmental factors water depth, SMGS and TOM. The analysis obtained optimal results, where 73.8% of total variance is explained by the first 2 axes (Fig 12). Based solely on the frequency of species, the dominant first axis almost completely represents water depth and the second axis shows the dependence on grain size as represented by vectors. The percentage of TOM is strongly correlated with water depth, simultaneously influenced in a weaker mode by decreasing grain size (Fig 12).

**Fig 13:**
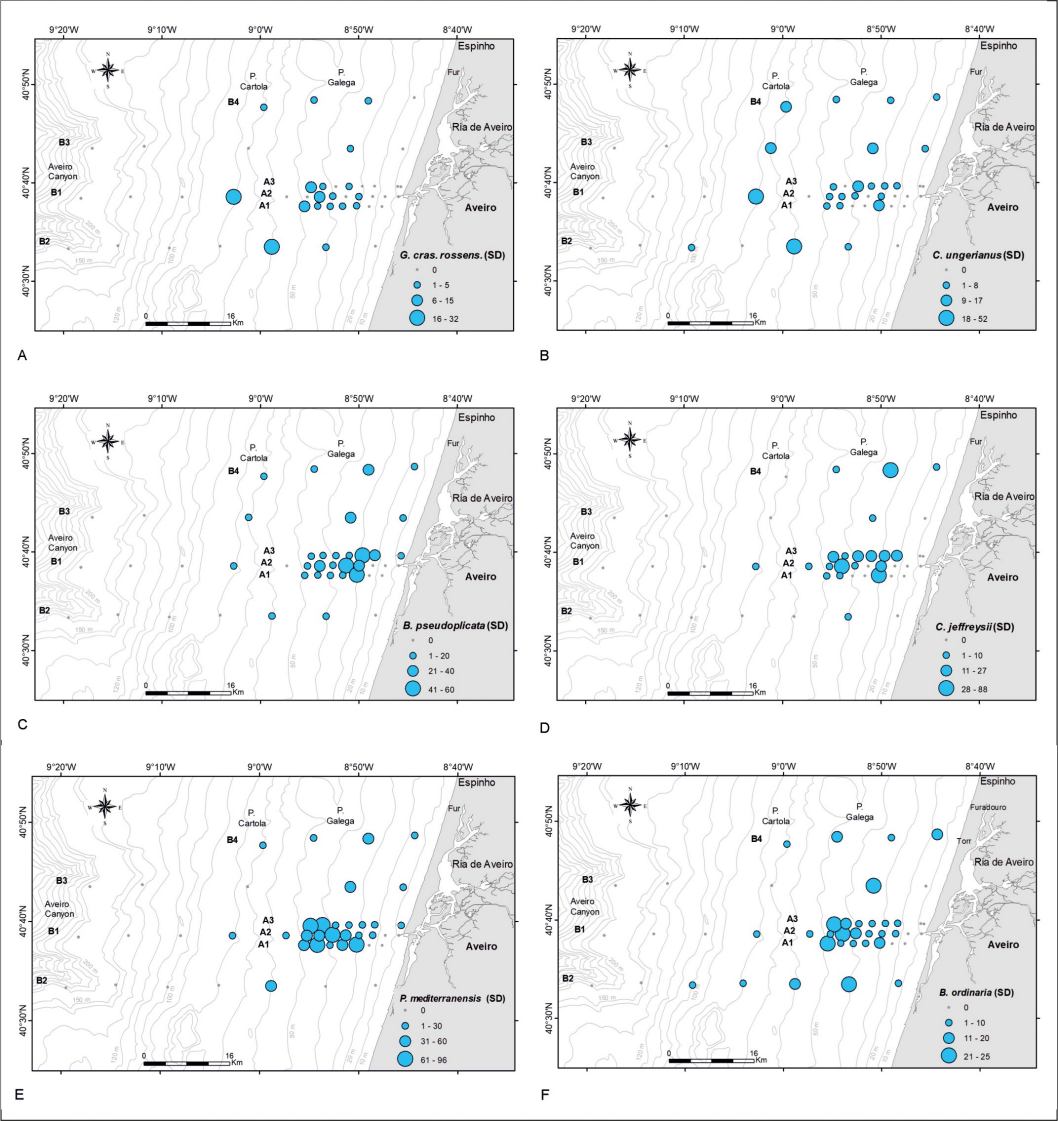
Distribution maps of the main species density of living organisms: A. *G*. *crassa rossensis* (*G*. *cras*. *rossens*.); B. *C*. *ungerianus*. *C*. *B*. *pseudoplicata*; D. *C*. *jeffreysii*; E. *P*. *mediterranensis*. F. *B*. *ordinaria*. Legend: Cartola—Pontal da Cartola; Galega–Pontal da Galega; Fur–Furadouro; VR—Vouga River. Adapted from Martins et al. [58].

**Fig 14:**
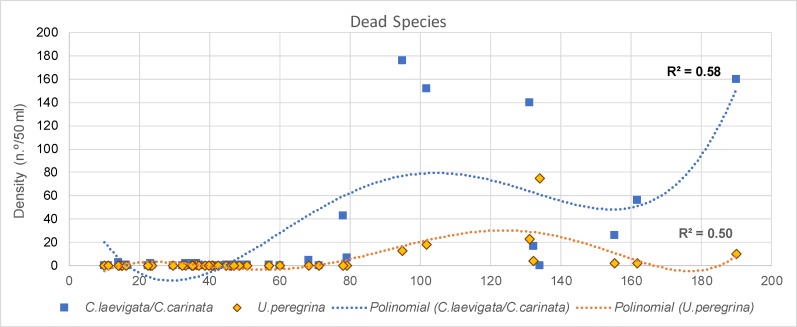
Density (SD; n.°/50 ml) of dead individuals of *C*. *laevigata*/*C*. *carinata* and *U*. *peregrina* as a function of the depth. Polynomial trend lines are presented.

Positioning frequencies of living individuals, represented in depth intervals, within CCA ordination provides interesting results. They start with low values at the first axis (shallow depths) and with medium values at the second axis (SMGS). The following two intervals (20–40 m and 40–60 m) do not follow the depth trend, but lean towards increasing grain size. Back to mean grain size, the interval 60–100 m shows a weak tendency to deeper sites followed by the deeper intervals 100–150 m and 150–200 m. The short stretching over the depth ordinate is caused by the low abundance of living individuals in the deep transect parts. Thus, distribution maps of the selected most abundant living species, related to the CCA groups 1 and 4–8 are presented in Fig 13. The CCA groups 2 and 3 contains species much more abundant in the DAs and a reduced number of individuals in the LAs. Since the species of each group have similar distribution patterns, the density of dead individuals of one species of each of these groups was plotted as a function of depth (Fig 14): *C*. *laevigata*/*C*. *carinata* (group 3) and *U*. *peregrina* (group 2). This plot evidences the abundance increasing of the of these species in the outer shelf.

Due to the much higher numbers of dead individuals, the succeeding intervals based on dead individuals follow the first axis representing water depth, when varying around medium ordinate values along the second axis indicating medium grain size. Only the shallowest interval (0–20 m) deviates from this trend being positioned in finer grain sizes (Fig 12).

**Fig 15:**
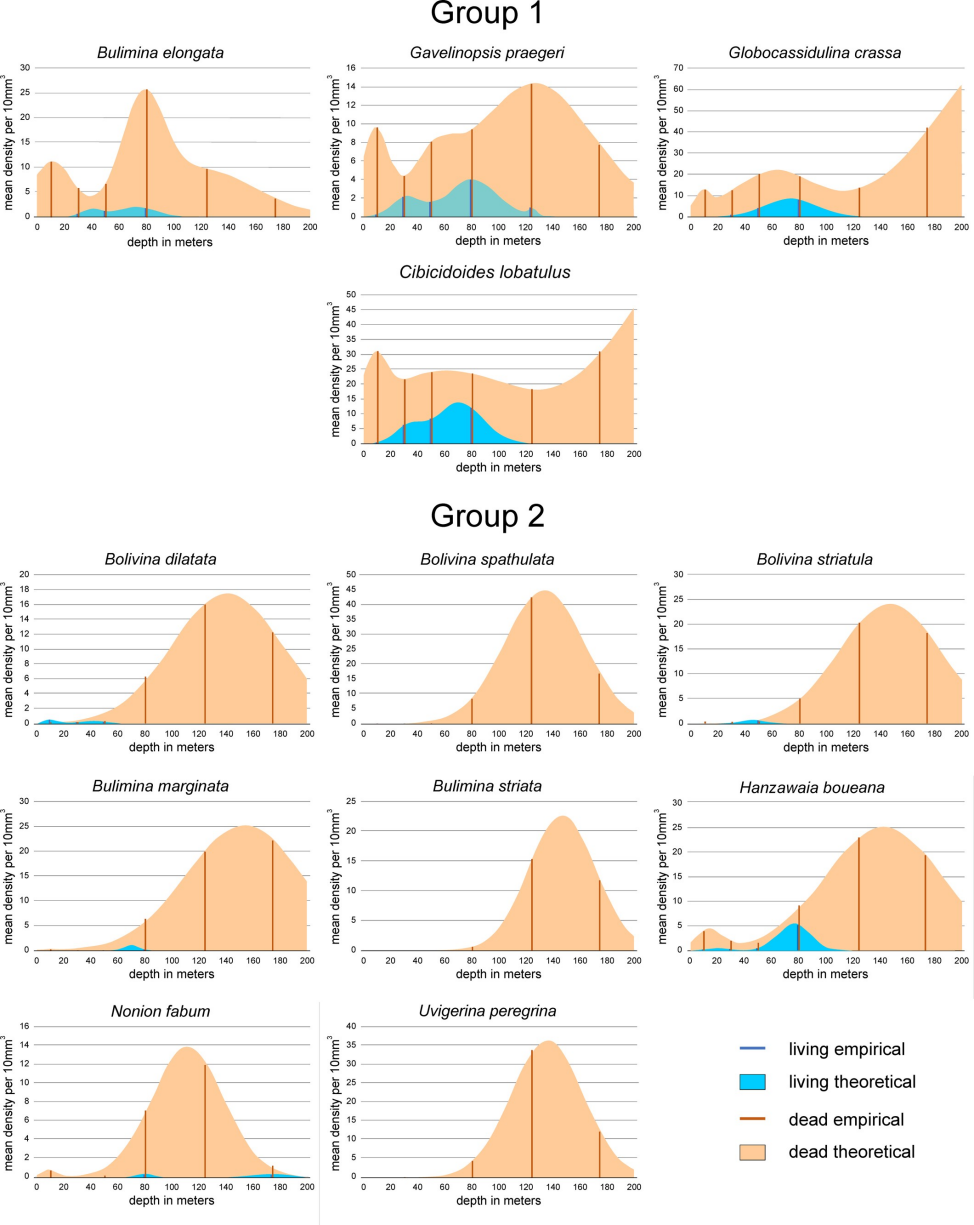
Depth frequencies of living and dead individuals for species of groups 1 and 2 according to UPGMA cluster analysis. Groups are positioned in the CCA of Fig 12.

**Fig 16:**
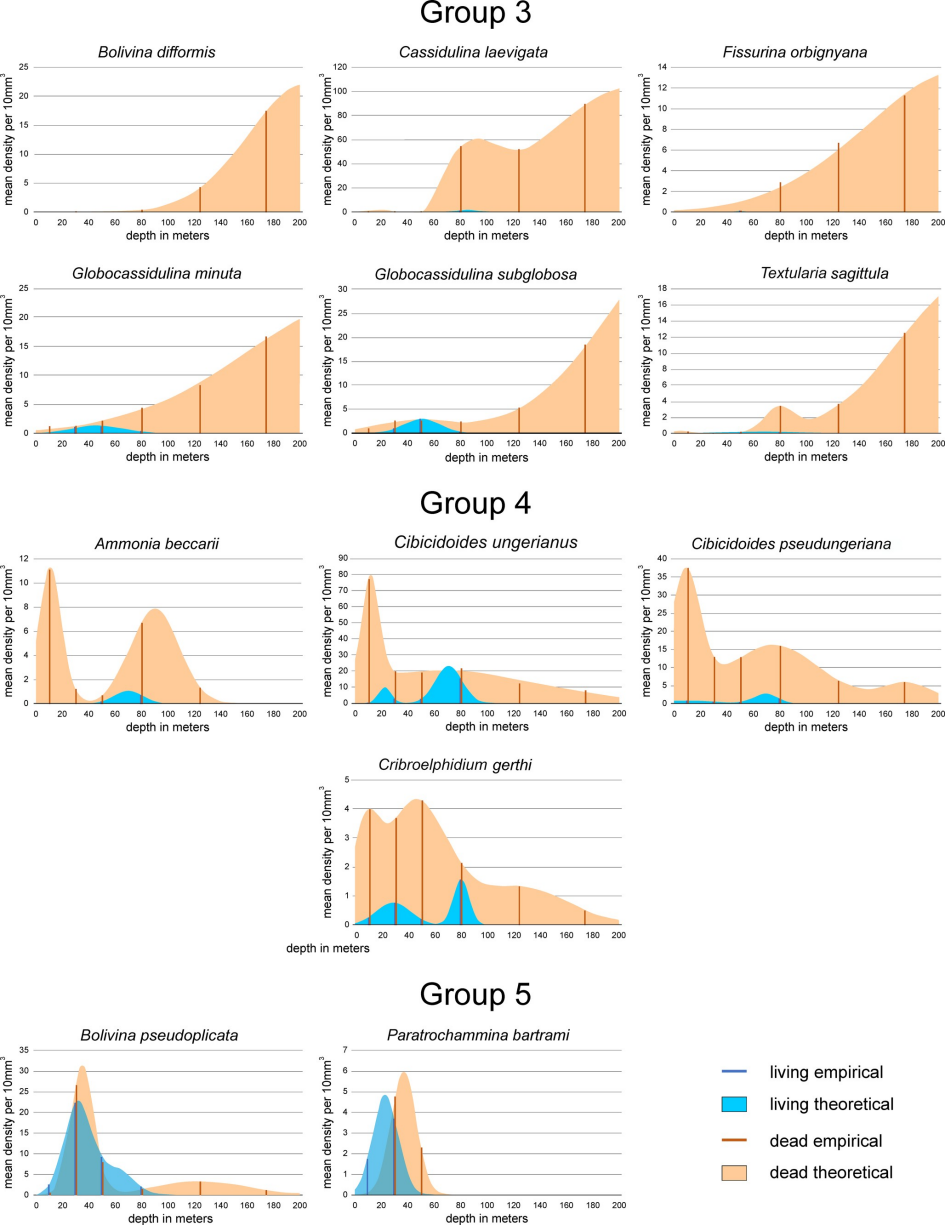
Depth frequencies of living and dead individuals for species of groups 3 to 5 according to UPGMA cluster analysis. Groups are positioned in the CCA of Fig 12.

**Fig 17:**
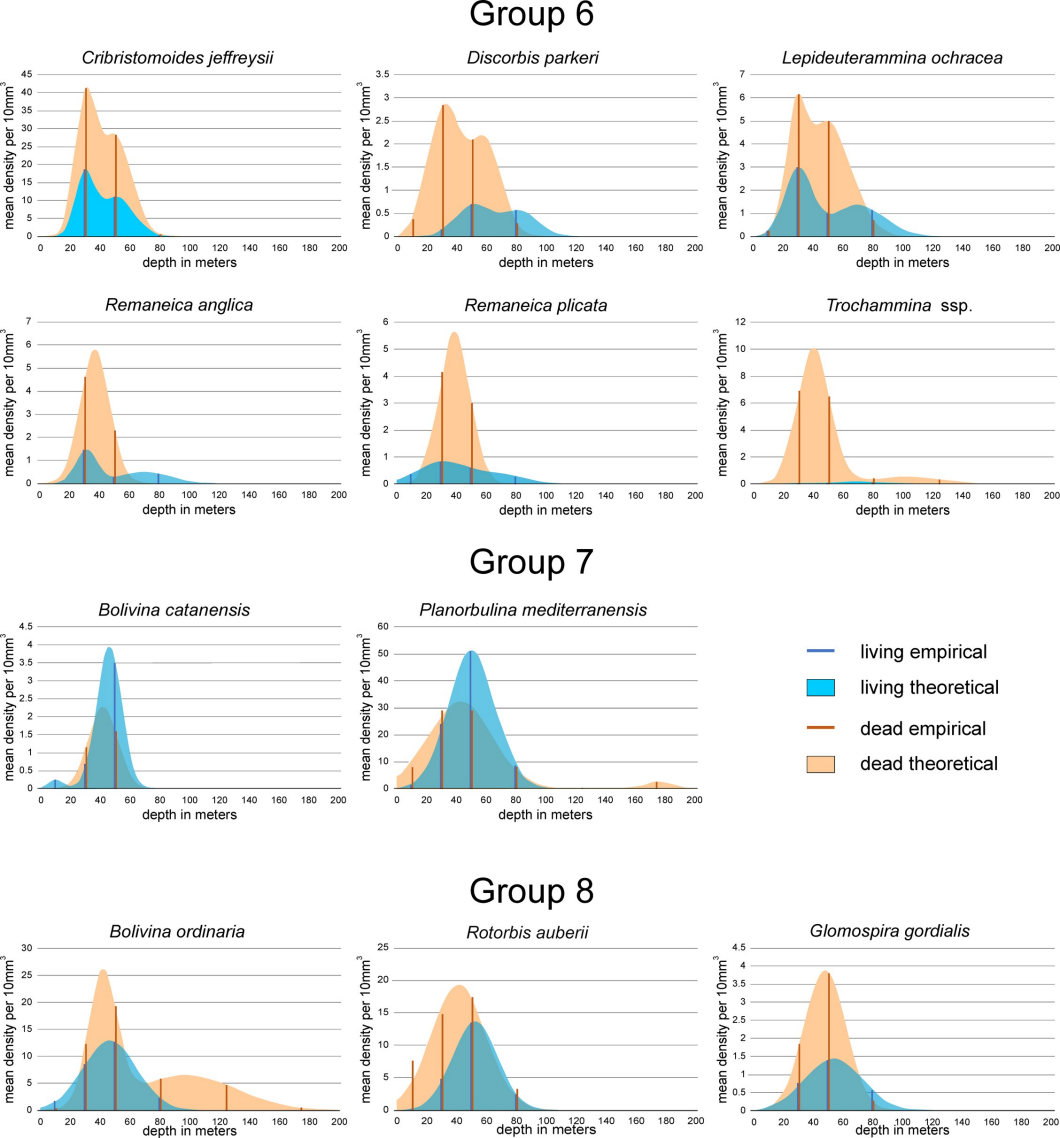
Depth frequencies of living and dead individuals for species of groups 6 to 8 according to UPGMA cluster analysis. Groups are positioned in the CCA of Fig 12.

Positions of species within the coordinate system gained by CCA could be explained by the frequency diagrams of species (Figs 15, 16 and 17). For an easier interpretation, diagrams are arranged according to groups resulting from Unweighted Pair Group Method with Arithmetic Mean (UPGMA) clustering based on Cosine measures that is independent from assemblage size. The distribution parameters ‘mean density’, the depth position of means in multimodal normal distributions and ranges expressed in the position of 0.5 and 99.5 percentiles are presented in Table 1.

**Table 1:** Distribution parameters for LAs and DAs of the most important species. The means of normal-distributed components are arranged according to their height, thus designated as modes. Groups indicated by colors given in Fig 12.

Living and Dead Species		Mean density	% Living individuals	Meters			Range in Meters	
				Mode 1	Mode 2	Mode 3	0.5 Percentile	95.5 Percentile
*Bulimina elongata*	living	1.8	4.9	72.5	40.1		21.1	110.1
	dead	13.3		77.5	118.3	10.9	0	224.6
*Gavelinopsis praegeri*	living	3.5	13.2	80.5	31.7	125.8	3.9	129.4
	dead	12.8		126.3	8.9	51.5	0	240.5
*Globocassidulina crassa rossensis*	living	8.5	9.1	73.5			24.0	132.1
	dead	77.9		249.5	61.1	8.5	0	409.7
*Lobatula lobatula*	living	12.4	15.0	70.8	33.1		7.4	116.1
	dead	51.5		246.5	8.5	60.7	0	390.0
*Bolivina dilatata*	living	0.4	0.8	10.0	41.3		0	66.7
	dead	17.5		140.7			36.7	244.8
*Bolivina spathulata*	living	0	0.0					
	dead	44.7		133.9			57.8	210.0
*Bolivina striatula*	living	0.7	0.6	46.6			25.9	67.3
	dead	24.2		146.9			50.7	243.2
*Bulimina marginata*	living	1.0	0.6	70.0			57.1	83.0
	dead	25.2		153.5	9.5		1.3	263.3
*Bulimina striata*	living	0	0.0					
	dead	22.6		146.8			83.4	210.2
*Hanzawaia boueana*	living	5.2	6.4	76.7	21.6		1.8	106.1
	dead	24.3		142.8	14.3	4.9	0	252.3
*Nonion fabum*	living	0.3	1.4	80.5	174.5		67.1	212.0
	dead	13.7		111.9	9.4		0	180.5
*Uvigerina peregrina*	living	0	0.0					
	dead	36.6		135.8			66.6	204.9
*Bolivina difformis*	living	0	0.0					
	dead	22.3		206.1			90.6	321.5
*Cassidulina laevigata/Cassidulina carinata*	living	1.9	0.4	85.5			44.2	95.8
	dead	98.8		207.8	84.5	21.5	20.2	367.6
*Fissurina orbignyana*	living	0.1	0.1	50.0			37.1	62.9
	dead	13.7		220.5			27.3	412.7
*Globocassidulina minuta*	living	1.3	4.4	46.5			0	98.3
	dead	23.0		249.8			15.5	484.5
*Globocassidulina subglobosa*	living	3.0	7.0	50.8			15.7	86
	dead	43.3		265.3	45.8		0	440.2
*Spiroplectammina sagittula*	living	0.1	1.1	68.6			0	137.4
	dead	18.8		228.0	79.3	5.1	0	372.8
*Ammonia beccarii*	living	1.1	5.0	70.0			42.0	98.1
	dead	9.2		11.1	90.4		0.0	137.7
*Cibicides ungerianus*	living	21.0	17.9	70.8	22.4		9.9	96.9
	dead	29.5		11.9	62.3		0	257.9
*Cibicidoides pseudoungeriana*	living	1.9	4.0	69.4	11.1		0	93.2
	dead	21.7		176.9	75.0	8.5	0	227.5
*Elphidium gerthi*	living	1.2	11.6	80.5	29.3		0	96.1
	dead	3.2		45.5	7.6	124.4	0	219.2
*Bolivina pseudoplicata*	living	18.1	79.7	31.7	60.7		4.7	94.5
	dead	22.7		35.1	124.3		12.1	217.5
*Paratrochammina bartrami*	living	4.8	80.2	23.4			0	48.3
	dead	5.9		37.0			11.8	62.2
*Cribrostomoides jeffreysii*	living	13.5	41.5	29.6	50.9		13.5	80.7
	dead	31.5		30.0	50.4		12.0	78.8
*Discorbis parkeri*	living	0.6	28.2	51.2	82.7		20.3	114.0
	dead	2.5		31.5	59.2		3.3	86.8
*Lepidodeuterammina ochracea*	living	2.1	49.6	30.1	69.9		6.3	115.1
	dead	4.7		28.6	49.0		8.0	90.1
*Remaneica anglica*	living	1.0	33.6	31.4	70.1		13.0	114.4
	dead	5.8		37.1			11.8	62.4
*Remaneica plicata*	living	0.7	32.8	30.1	67.8		0	113.6
	dead	5.6		38.8			11.8	65.6
*Trochammina* spp.	living	0.2	2.8	69.8	32.7		12.3	115.6
	dead	9.0		40.1	99.3		11.9	167.3
*Bolivina catanensis*	living	3.8	153.1	45.8	10.0		0.0	67.6
	dead	2.3		41.6			16.0	67.2
*Planorbulina mediterranensis*	living	51.2	116.3	50.2			9.1	91.3
	dead	31.2		42.8	175.5		0	201.2
*Bolivina ordinaria*	living	12.9	51.1	46.4			0	93
	dead	15.4		41.8	96.5		17.2	185.5
*Discorbis mira*	living	13.6	59.2	52.3			13.3	91.3
	dead	19.3		41.0			0	88.0
*Glomospira gordialis*	living	1.4	51.2	53.6			3.4	103.9
	dead	3.9		47.8			11.4	84.3

Group 1: This group consisting of *Bulimina elongata*, *Gavelinopsis praegeri*, *Globocassidulina crassa rossensis* and *Cibicidoides lobatulus* is positioned in the center of the CCA coordinate system (Fig 12) and represents the transition between the shallow-water groups 4 to 8 and the deeper-water groups 2 and 3. Living individuals are abundant in *C*. *lobatulus* and *G*. *crassa rossensis* with mean density peaks (MDPs) around 10 individuals, and less abundant in *G*. *praegeri* and *B*. *elongata* with MDPs around 2 individuals (Fig 15; Table 1). The prominent 1^st^ mode is located in all species around 75 m. Depth ranges start in *G*. *praegeri* and *C*. *lobatulus* within the first 10 m, while in *G*. *crassa rossensis* and *B*. *elongata* between 20 m to 25 m up to 110 m and 135 m. Similar to living individuals, dead specimens are abundant in *G*. *crassa rossensis* (MDP: 77.9) and *C*. *lobatulus* (MDP: 51.5), while less abundant in *G*. *praegeri* (MDP: 12.8) and *B*. *elongata* (MDP: 13.3). The trimodal frequency distributions differ in the succession of modes. The shallowest mode means are located in all species around 9 m, failing to become the most prominent mode (Fig 15; Table 1). The second mode, deeper means are located in all species between 50 m and 80 m, becoming the first mode in *B*. *elongata*. The third one means show a differentiation between *B*. *elongata*, *G*. *praegeri* and *G*. *crassa rossensis*, *C*. *lobatulus*. Located around 120 m for the former species group, they are positioned around 250 m in the later species, becoming here the most prominent peaks. Depth ranges start in all species at 0 m caused by the truncated normal distributions with the first mean. They finish for *B*. *elongata* and *G*. *praegeri* between 220 m and 250 m, while for *G*. *crassa rossensis* and *C*. *lobatulus* the 99.5 percentiles are located deeper than 300 m. This shape hints to an additional normal-distributed component stretching the distribution of dead individuals into deeper regions. Percentages of living individuals on dead specimens range from 4.9% to 15.0% (Table 1).

Group 2: *Bolivina dilatata*, *Bolivina spathulata*, *Bolivina striatula*, *Bulimina marginata*, *Bulimina striata*, *Hanzawaia boueana*, *Nonion fabum* and *Uvigerina peregrina* are members of this group (Fig 15, Table 1). Living individuals are rare in *H*. *boueana* (MDP 5.2), extremely rare with MDPs < 1.0 in *B*. *dilatata*, *B*. *striatula*, *B*. *marginata* and *N*. *fabum*, and completely lacking in *B*. *spathulata*, *B*. *striata* and *U*. *peregrina*. In *B*. *marginata*, *H*. *boueana* and *N*. *fabum*, the main mode is located around 75 m, different to the shallower positions of *B*. *striatula* (46.6 m) and *B*. *dilatata* with 10 m (Table 1). Depth ranges of living individuals start near the surface (*B*. *dilatata*, *H*. *boueana*), at 26 m (*B*. *striatula*) and around 62 m (*B*. *marginata*, *N*. *fabum*). Depth ranges cover between 66.7 m (*B*. *dilatata*) and 212 m (*N*. *fabum*). Dead specimens of all species are characterized by a main peak positioned between 111.9 m (*N*. *fabum*) and 153.5 m (*B*. *marginata*) characterized by MDPs between 17.5 (*B*. *dilatata*) and 44.7 individuals (*B*. *spathulata*). Except *B*. *marginata*, *H*. *boueana* and *N*. *fabum* possessing additional components, the first mode is the singe peak for the remaining species within this group (Table 1). Depth ranges start near the surface in *B*. *marginata*, *H*. *boueana* and *N*. *fabum*, followed with onsets from 36.7 m (*B*. *dilatata*) to 84.3 m (*B*. *striata*). Except *N*. *fabum* possessing a 99.5 percentile of 180.5 m, depth ranges finish between 200 and 270m (Table 1). Percentages of living individuals on dead specimens are between 0 and 1%, except *N*. *fabum* (1.4%) and *H*. *boueana* (6.4%; Table 1).

Group 3: *Bolivina difformis*, *Cassidulina laevigata*, *Fissurina orbignyana*, *Globocassidulina minuta*, *Globocassidulina subglobosa* and *Textularia sagittula* belong to this group. Living individuals are extremely rare with MDPs from 0.1 to 3.0 individuals or being absent (*B*. *difformis*). The single modes are located between 46.5 and 85.5 m (Fig 13, Table 1). Depth distributions start either at 0 m (*G*. *minuta*, *T*. *sagittula*) or between 15 and 45 m in the remaining species, except *B*. *difformis*. All distributions end between 60 and 140 m (Table 1). Distributions of dead individuals are unimodal (*B*. *difformis*, *F*. *orbignyana*, *G*. *minuta*), bimodal (*G*. *subglobosa*) or trimodal (*C*. *laevigata*, *T*. *sagittula*), with MDPs between 13.7 in *F*. *orbignyana* and 23 individuals in *G*. *minuta*. High (43.3) and extreme (98.8) numbers of individuals characterize the MDPs of *G*. *subglobosa* and *C*. *laevigata*. The first modes of all species are located below 200 m depth, thus outside the investigated transect. Depth distributions start at the surface (*G*. *subglobosa*, *T*. *sagittula*) or between 15 m and 30 m in the remaining species, except *B*. *difformis* that starts at 90.6 m. The deeper limits are located beyond 300 m depth; thus, a further deeper normal-distributed component must be expected for all species in this group. The percentages of living individuals on dead specimens are low, similar to group 3, with slightly higher values for *G*. *minuta* (4.4%) and *G*. *subglobosa* (7.0%).

Group 4: *Ammonia beccarii*, *Cibicidoides ungerianus*, *Cibicidoides pseudoungeriana* and *Cribroelphidium gerthi* are taxa belonging to this group. Their positions in CCA indicate shallow depths and finer grain size (Fig 12). MDPs are low (1 to 2 individuals) in *A*. *beccarii*, *C*. *pseudungeriana* and *C*. *gerthi*, becoming more abundant in *C*. *ungerianus* (21 individuals). Depth distributions are unimodal (*A*. *beccarii*) or bimodal in the other species with the characteristic first mode located between 69.4 m and 80.5 m (Fig 16, Table 1). Depth distributions start at the surface (*C*. *pseudoungeriana*, *C*. *gerthi*) or at 10 m (*C*. *ungerianus*) or 50 m (*A*. *beccarii*). All distributions end between 90 and 100 m. Dead individuals show low MDPs in *C*. *gerthi* (3.2) and *A*. *beccarii* (9.2), becoming higher in *C*. *pseudoungeriana* (21.7) and *C*. *ungerianus* (29.5). Distributions are bimodal (*A*. *beccarii*, *C*. *ungerianus*) or trimodal (*C*. *pseudoungeriana*, *C*. *gerthi*) with changing positions of the most important mode 1. Shallow positions of the first mode around 11.5 m are characteristic for *A*. *beccarii* and *C*. *ungerianus*, while deeper positions can be found in *C*. *gerthi* (45.5m) and *C*. *pseudoungeriana* (176.9 m). All depth distributions start at the surface and finish, except *A*. *beccarii* (137.7m), between 200 m and 260 m (Fig 16, Table 1).

In contrast to the species of groups 2 and 3 indicating deeper environments, percentages of living individuals on dead specimens are higher, still low in *C*. *pseudoungeriana* (4.0%) and *A*. *beccarii* (5.0%), but higher in *C*. *gerthi* (11.6%) and *C*. *ungerianus* (17.9%). Altogether, this explains the similarities in distributions of species belonging to group 1 and group 4, primarily expressed in living individuals (Figs 14 and 15).

Group 5: The abundant *Bolivina pseudoplicata* and the rare *Paratrochammina bartrami* are members of this group. They differ in living individuals by MDPs of 18.1 and 4.8 individuals (Table 1). Furthermore, distributions are unimodal in *P*. *bartrami* and bimodal in *B*. *pseudoplicata* (Fig 16). The prominent modes 1 are located around 25 m. Both species start at or near the surface, but differ in their distribution’s end (*P*. *bartrami*: 48.3 m; *B*. *pseudoplicata*: 94.5 m). Dead specimens are also rare in *P*. *bartrami* (MDP 5.9) and more frequent in *B*. *pseudoplicata* with a MDP of 22.7. Both MDPs are slightly higher than their counterparts in living individuals (Table 1). The main modes in the unimodal distribution of *P*. *bartrami* and the bimodal distribution of *B*. *pseudoplicata* are located around 36 m. Depth distributions range from 11.8 m to 62.2 m in *P*. *bartrami* and from 12.1 to 62.2 m in *B*. *pseudoplicata*. Although their frequencies are different, the proportions of living individuals on dead specimens are the same (79.7% and 80.2%; Table 1).

Group 6: *Cribrostomoides jeffreysii* is the most abundant member in this group, while *Discorbis parkeri*, *Lepideuterammina ochracea*, *Remaneica anglica*, *Remaneica plicata* and *Trochammina* ssp. are rather rare. In living individuals this is marked by MDP of 13.5 individuals for *C*. *jeffreysii*, strongly different from MDPs of the other group members ranging from 0.2 to 2.1 (Table 1). Distributions of living individuals are bimodal with an almost shallower mode 1 approximately located between 30 and 50 m, and a second mode positioned between 50 and 80 m. Only the few living *Trochammina* ssp. is opposite with a deeper mode 1 compared to mode 2 (Table 1). Depth distributions start near the surface in *R*. *plicata* and *L*. *ochracea*, or are positioned between 12 m and 20 m in the other species. The distribution ends for *C*. *jeffreysii* at 80.7 m, while for the other species distributions end in a narrow interval between 113.6 m and 115.6 m (Table 1). Frequencies of dead individuals are similar to living individuals, with medium abundance in *C*. *jeffreysii* (MDP 31.5) and low abundance (MDP 2.5 to 9.0) in the other species (Table 1). Depth distributions are unimodal in both *Remaneica* species, and bimodal in the other. The first modes are similarly located between 28.6 m and 40.1 m, followed by second modes positioned between 49 m and 60 m, except *Trochammina* ssp. with a mode 2 of 99.3 m. Depth ranges start between 8 m and 12 m (except *D*. *parkeri* starting close to the surface) and end between 60 m and 90 m (except *Trochammina* ssp. with deeper limits). Percentages of living individuals on dead specimens are similar being between 28.2% and 41.5%. Only *Trochammina* ssp. shows lower proportions (2.8%).

Group 7: The rare *Bolivina catanensis* and the abundant *Planorbulina mediterranensis* as members of this group show similar distribution forms for both living and dead individuals (Fig 17). The MDP of *B*. *catanensis* is low (3.8) compared to *P*. *mediterranensis* (51.2), but positions of the first mode are similar (45.8 m *vs*. 50.2 m). The restricted depth ranges start near the surface, ending at 67.6 m and 91.3 m, respectively (Table 1). Dead individuals show lower abundance compared to living individuals in both species, where the MDP of *B*. *catanensis* is again low (2.3) compared to *P*. *mediterranensis* (31.2). Although positions of the first modes are nearly identical (41.6 m and 42.8 m), *P*. *mediterranensis* shows a strong second component characterized by mode 2 located at 175.5 m (Fig 17; Table 1). Distribution limits are narrow for *B*. *catanensis* (16 m to 67.2 m) and wide for *P*. *mediterranensis* (0 to 201.2 m). In face of strong abundance differences between both group members, the percentages of living individuals on dead specimens exceed 100% in both specimens (Fig 17; Table 1), which is the main characteristic for this group.

Group 8: The abundant *Bolivina ordinaria*, *Rotorbis auberii* and the less abundant *Glomospira gordialis* belong to this group (Fig 17, Table 1). Living individuals possess MDPs of 12.9 and 13.6 in *B*. *ordinaria* and *R*. *auberii*, while the MDP of *G*. *gordialis* is much lower (1.4). The unimodal distributions similarly peak between 45 m and 55 m (Table 1). Depth distributions start near the surface or at 10 m and end around 100 m. The distribution of dead individuals is similar to the living forms with high MDPs for *B*. *ordinaria* and *R*. *auberii* (15.4, 19.3) and a low MDP for *G*. *gordialis* (3.9). First modes of the unimodal *R*. *auberii* and *G*. *gordialis* and of the bimodal *B*. *ordinaria* are located within a small interval between 41 m and 48 m. Depth distributions start near the surface in *R*. *auberii* and around 15 m in the other species. They end in the unimodal *R*. *auberii* and *G*. *gordialis* at 88 m and 84.3 m. The distribution of the bimodal *B*. *ordinaria* stops at 185.5 m (Fig 17, Table 1). Percentages of living individuals on dead specimens are high and range from 51.1 to 59.2%.

## Discussion

### Distribution of benthic foraminiferal species in LAs and DAs

The composition of benthic foraminiferal assemblages in the study area is typical of a temperate marine realm [56,72–73] with temperatures of ≈14.5°C near the coast and <14ºC at depths >25 m, decreasing to 12.5ºC at the shelf break (S1A Fig). Bottom salinities are ~36.1–36.2 in the shallowest stations and decrease down to 35.4 near the lagoon mouth and to ≈36.3 on the shelf break (S1B Fig). The water densities vary from 25.4 to 26.4 at the surface but are higher than 27.0 below 50 m (S1C Fig). In this type of environment, a rich foraminiferal community was found both in LAs and DAs (S2 Table), though with distinct patterns of density, diversity and species distribution. The CCA (Fig 12) allowed to recognize 8 groups of species in which the divergence of LAs and DAs as a function of depth has been analyzed.

Group 1 is represented by *B*. *elongata*, *G*. *praegeri*, *G*. *crassa rossensis* (Fig 13A) and *C*. *lobatulus*. These species are relatively abundant in DAs where *G*. *crassa rossensis* and *C*. *lobatulus* tend to increase in abundance in deeper areas, whereas *B*. *elongata* and *G*. *praegeri* show an opposite trend (Fig 15). A similar distribution pattern was described by Levy et al. [55] for *B*. *elongata* and *G*. *praegeri* at the PCS, where these species are common from the coast up to 150 m, but rarer beyond this depth. However, a caveat is made; these authors analyzed the total (living + dead) foraminiferal assemblage. In LAs of ACS, *B*. *elongata*, *G*. *praegeri*, *G*. *crassa rossensis* and *C*. *lobatulus* exhibit mean or low abundance and occupy biotopes located between 50 and 80 m. The LAs belonging to Group 1 following the pattern of distribution of *G*. *crassa rossensis* (Fig 13A) is restricted in a mid-shelf biotope characterized by stable substrate, not constantly mixed and stirred by the winds, waves and tides, and is located out of the direct disturbance caused by the Ria de Aveiro outflow. The DAs instead cover a much broader depth range than the LAs, suggesting that the DAs may be remobilized by waves and dispersed by oceanic currents for a large area and/or living populations of these species may inhabit other sectors of the PCA.

Group 2 is represented by *U*. *peregrina*, *B*. *spathulata*, *B*. *marginata*, *H*. *boueana*, *B*. *striatula*, *B*. *striata*, *B*. *dilatata* and *N*. *fabum* (Fig 12). These taxa are associated with finer grained substrates and high TOM content (Fig 12) and are mostly represented in DAs from the deeper sectors of the ACS, as shown in the plot of *U*. *peregrina* density in DAs as a function of depth (the species selected to represent this group; Fig 14) and also in Fig 15. This sector (80–120 m depth) is characterized by low degree of disturbance by hydrodynamic forces and variability of physicochemical parameters. This group includes species common in neritic and bathyal depths in productive coastal upwelling areas and in oxygen-poor environments [73–82].

Group 3 is characterized by *B*. *difformis*, *C*. *laevigata*, *G*. *subglobosa*, *G*. *minuta*, *F*. *orbignyana* and *T*. *sagittula*. These taxa reach relatively high densities in DAs and are extremely rare or absent (*B*. *difformis*) in LAs (Fig 16). The density of these species, mostly on DAs, also tends to increase with depth and TOM (Fig 12). In DAs, these species are present from the shallowest stations (except *B*. *difformis*) but show the highest densities at 200 m depth (as can also be observed in the plot of *C*. *laevigata*/*C*. *carinata* density, as a function of depth, the taxa selected to represent the pattern of this group in DAs; Fig 14). Most of these species are related to pulses of food supplied by the oceanic productivity [56,79,83–86].

Group 4 includes *C*. *ungerianus* (Fig 13B, in LA) and species that follow similar pattern of distribution such as *A*. *beccarii*, *C*. *pseudungeriana* and *C*. *gerthi*. In ACS, these taxa are associated with finer grained and common at shallow depths and at the mid shelf sediments both in LAs and DAs, but end their distributions between 90/100 m in LAs and 140/200 m in DAs (Figs 12 and 16). *Cibicidoides ungerianus* is an epifaunal species [72,73] that lives in oxic environments [87–88] with no tolerance to oxygen deficiency [82]. Living populations of *C*. *gerthi* mainly depend on the availability of food, particularly diatoms [89]. The highest density of the species of this group should be related with availability of food of high quality and oxic environments.

Group 5 encloses *P*. *bartrami* and *B*. *pseudoplicata* (Fig 13C, in LA). This group is associated with fine grained sediment and occurs in shallow waters of ACS (Fig 12). These taxa have abundance peaks around 25 m in LAs and 36 m in DAs of ACS (Fig 16). While *P*. *bartrami* ends its distribution at 62.2 m, *B*. *pseudoplicata* occurs at all depths at least in DAs (Fig 16) and has been reported from the infralittoral to bathyal environments [76,90–91]. *Bolivina pseudoplicata* is much more abundant than *P*. *bartrami*, both in LAs and DAs (Fig 16). Due to their opportunistic behavior and tolerance to low salinity [92–93] both species are recorded in ACS near the Ria de Aveiro mouth associated with a certain degree of disturbance caused by the organic-enriched outflow from the lagoon and the variability of physicochemical parameters.

Group 6 comprises *C*. *jeffreysii* (the most abundant), *D*. *parkeri*, *L*. *ochracea*, *R*. *anglica*, *R*. *plicata* and *Trochammina* ssp. (rare species) (Fig 17). In both assemblages, these species are related to relatively coarse sediments (Fig 12). The LAs of *D*. *parkeri*, *L*. *ochracea*, *R*. *anglica*, *R*. *plicata* and *Trochammina* ssp. have reduced density, whereas *C*. *jeffreysii* is quite well represented (Fig 13D, in LA). Their abundances in DAs is higher than the LAs but demonstrate similar distribution patterns with the first shallow modes almost coincident in both assemblages (Fig 17). *Cribrostomoides jeffreysii* can be found on all PCS down to 90/150 m [56] but in ACS its highest density is mostly identified in front of the Aveiro lagoon outflow, associated with coarse grain size both in LAs (Fig 13D) and DAs (Fig 17). Most of the species of this group are epifaunal and oxyphilic and can tolerate low salinities [94] that agrees with their pattern of distribution in ACS (Fig 17).

Group 7 includes *P*. *mediterranensis* (the most abundant; Fig 13 E, in LA) and *B*. *catanensis* (present with reduced abundance). Both species occupy similar ranges of depth in both LAs and DAs (Fig 17) and are associated to coarse grained sediments (Fig 12). They rarely occur at shallower stations but display their abundances’ peak in the mid shelf (at 45.8 m and 50.2 m depth, respectively) and disappear at 67.6 m and 91.3 m, respectively. *Planorbulina mediterranensis* is common (> 6%) in the PCS down to 75 m, becomes rare at depths greater than 150 m and disappears beyond the continental shelf break [56]. Sediment grain size is a limiting factor for *P*. *mediterranensis* that gets its maximum abundance on coherent rock or at coarse sediments containing gravel and sand [73]. It is an epiphytic species that can live attached by the spiral side to plants and rocks [95–96]. Thus, it has been associated with coarse grained sediments of the mid ACS, in front of the Ria de Aveiro mouth, where it can also benefit of organic matter supplied by that lagoon. The reduced density of *B*. *catanensis* and *P*. *mediterranensis* in DAs in comparison with the living one might indicate tests removal by hydrodynamic forces in the mid ACS.

Group 8 includes *B*. *ordinaria*, *R*. *auberii* (both abundant) and *G*. *gordialis* (less abundant) (Fig 12). In ACS, the LAs of these species are present from the shallower stations until ≈100 m depth and have similar unimodal distributions with peaks at the mid shelf between 45 m and 55 m, associated with the coarsest sediments (Figs 12 and 17). The ranges of depth of the DAs are similar in the study area and exhibit abundances’ peaks from 30 to 50 m depth. *Rotorbis auberii* is an epifaunal species quite common in the northern region of the Iberian Peninsula [97–98] as well as *G*. *gordialis* [56]. The map of distribution of *B*. *ordinaria* is selected to represent this group in LAs (Fig 13F) evidences higher densities mainly in the sector between 40–60 m deep. *Bolivina ordinaria* is highly adaptable, has a wide tolerance to different environmental parameters leading to a ubiquitous distribution [9] but its abundance increases in zones with high input of fresh phytoplankton [9]. In ACS, the LAs of *R*. *auberii* and *G*. *gordialis* show a wider depth range than the DAs. Both assemblages are mainly present at the mid shelf gravel deposits, where the accumulation of fine particles is reduced. The contraction or lag of the dead faunas of these species in ACS (Fig 17) may also indicate removal of tests by hydrodynamics.

The distribution of the main species and the groups identified by the CCA indicate the existence of several sub-environments in ACS depending probably on the greater or lesser variability of the physicochemical parameters, the hydrodynamism, the stability of the substrate, the type of availability of organic matter.

### Differences in the structure of LAs and DAs of benthic foraminifera

Densities of DAs reach, in general, higher values than LAs, and tend to increase at deeper stations, where the number of living organisms are quite low during sampling events (Fig 3A and 3B). At the shallowest stations (≈10–15 m) both living and dead foraminiferal densities are low (Fig 3B).

#### LAs and DAs at the inner shelf

The low rank distances, at depths 15–25 m, are related to low ‘Incorporation Values’ (Figs 5 and 6) and are associated to TOM contents of <0.5%, which means that the living organisms are contributing with few empty tests for the sedimentary record. Moreover, the Diversity/Heterogeneity diagrams show that in the shallowest sites (~10 m) of A-transects, species richness is low combined with high heterogeneity in DAs, while LAs are absent or have the same diversities as DAs (Figs 8 and 9). Diversity diagrams of sites in the B-transects show similar configurations at 15 m comparable to sites in the A-transects at 10 m, while diagrams in the A-transects at 15 m are devoid of living individuals and high species richness combined with low heterogeneity in DAs (Fig 9). These characteristics associated to a low density of foraminifera (Fig 3), particularly close to the Ria de Aveiro mouth and in some places of the transepts B, indicate the occurrence of high environmental disturbance.

This inner shelf area is affected by the deposition of sediments supplied from the mainland to the oceanic system [99]. In the shallower zones, detrital particles and empty tests of foraminifera should be resuspended by waves and transported by coastal drift currents parallel to the coastline. Moreover, the shallowest stations of A-transects located up to 30 m may be affected by the deposition of sediments associated with the ebb tide delta of the Ria de Aveiro, where sediments resulting from the lagoon and the coastal drift are deposited [100]. The influence of this sedimentary structure should be extended until 25/30 m deep in front of the Ria de Aveiro mouth [100].

The dynamic of other morphological features common in the region, such as the longshore bars that are elongated and sub-parallel to the coast-line and correspond to important accumulation of sediments [101] also may affect the dimension and structure of the living benthic communities and the preservation of dead ones. The magnitude and dynamics of these structures depend on meteorological and hydrodynamic conditions and the oceanographic regime [101]. They may affect the stability of the sediment generating unfavorable environments for the establishment of LAs and contributes to the burial and destruction of empty tests. As observed by Dimiza et al. [22], the prevailing environmental conditions, such as hydrodynamics, vegetation cover and fresh water influx, may have strong impact on the taphonomic processes.

In the northern PCS, the theoretical evaluation of the remobilizer and transporter potential of waves and currents was carried out for instance by [49,52,101–103]. According to these authors, the wave actions are one of the main, if not unique, mechanisms with the ability to remobilize particles, with currents acting as transport engines for particles suspended by the waves. During periods of stormy weather, the silt and very fine to fine sand deposited at the continental shelf bottom are remobilized with frequencies that decrease as the depth increases [49,52,101–103]. Thus, in the shallower zone of ACS, the strong hydrodynamics, which cause the frequent remobilization of sediments coupled with changes of physicochemical factors, should prevent the development of large living benthic communities and the accumulation of empty tests of dead foraminifera. The advective transport of materials from the Douro River [104], the coastal erosion at north of the Aveiro parallel due to the longshore currents (coastal drift) and the Ria de Aveiro contribution [49,100] may “dilute” and bury the empty tests of foraminifera in the shallower zone of ACS. In the inner shelf, the sedimentary dynamic and the hydrodynamics limit the accumulation of materials with low density (such as detritic and biogenic particles as well as particulate organic matter), and both living and dead organisms have therefore reduced abundance. According to Taborda [103] fine to very fine sands deposits of the inner continental shelf, up to 30 m depth, are commonly remobilized (40% of the time over a year), with moderate to high agitation conditions that are the most conducive to sediment transport. Therefore, sediment instability seems to be one of the most unfavorable factors to the development of large living foraminiferal communities and the active sedimentary processes seems to be unfavorable to the accumulation of tests after the organism’s death in the inner shelf.

#### LAs and DAs at the mid/outer shelf

The comparison of the standing crops of LAs and DAs (densities based on 10 cm^3^ sediment) through the Spearman rank distances show that the values of this variable increase from shallower stations until 50 m where it reaches a maximum (Fig 5) and where the IncorpVals are higher (Fig 6). Between ≈25–80 m depth (Fig 5), rank distances increase as associated mostly to higher ‘Incorporation Values’ (Fig 6) and SimDivers’ between LAs and DAs. The coincidence of diversity diagrams in LAs and DAs (Figs 8 and 9) is remarkable for sites located in all B-transects between 35 m and 60 m and in some sites of A-transects (A3-5, A3-8) in the same depth range and some stations between 60 m to 80 m (B1-2, B2-3 and B4-4). The SimDivers’ values reached maximum values (SimDivers = 65) at 1350 μm and at 0.55% TOM. These results indicate that this zone is in general more favorable to the establishment of large standing crops of LAs; living communities tend to be more diversified contributing a larger number of empty tests to the sedimentary record, whose record seems to be lost later.

Several sub*-*environments were identified in the mid to outer shelf region. For example, in front of the lagoon mouth, in most of the stations of the transects A located between 20–60 m, the “receiving>accordance” or “receiving≈accordance” and the TOM contents reached values >1.5%, indicating significant contribution of organic matter from the Ria de Aveiro to adjacent continental area. In this region between 20–40 m with relatively coarse sediments, an oxyphilic LA tolerant to low salinities composed mostly by *C*. *jeffreysii* and several epifaunal species such as *D*. *parkeri*, *L*. *ochracea*, *R*. *anglica*, *R*. *plicata* and *Trochammina* ssp. (Figs 12 and 17; Group 6) and between 40–60 m a LAs including mostly *P*. *mediterranensis* (the most abundant, Fig 13E, in LA) and *B*. *catanensis* (Figs 12 and 17; Group 7) are found. The most opportunistic species of the 20–40 m-LA seems to be *C*. *jeffreysii* and in the 40–60 m LAs should be *P*. *mediterranensis* that may also be the ones that most contributes with tests to the DAs. The IncorpVals in this region indicate that surface sediments have newly developed LAs and constitute a very recent memory of the previous standing crop of LAs (DAs) still unchanged (at least significantly) by the effects of sedimentary dynamics.

Another sub-environment identified in the mid to outer shelf region is characterized by the rank distances between LAs and DAs with the highest degree of deviation for both assemblages at SMGS of ~1600 μm (Fig 5). This region is essentially located between 40-60m, and both LAs and DAs are characterized by the occurrence of species such as *B*. *ordinaria*, *R*. *auberii* (both abundant) and *G*. *gordialis* (Figs 12 and 17; Group 8). Relatively high densities of living individuals are remarkable in transects A but also in transects B2 and B4 (Fig 3A; S3 Table), at the region where the substrate is composed by gravel deposits (between 30 m and 50 m). Indeed, in few stations (B4-2, B2-2, B2-3) of this sector, densities of living foraminifera reached higher values than the dead ones. This zone is outside the influence of the strongest hydrodynamic processes and active sediment dynamics of the inner shelf. The IncorpVals reach the highest values indicative of a higher contribution of LAs standing crops to the DAs (Fig 6). The production of tests seems to be highest mostly under the influence of the lagoon outflow (in stations of A-transects) where the “receiving” is higher than the “accordance” (Fig 6), but the high production of tests by living organisms does not revert to a corresponding increase in dead association in the mid shelf gravel deposits. In fact, the presence of gravely deposits at mid ACS (30 m to 80 m depth) suggests that this region, out of the main influence of the alongshore transport of sediments, probably receives a smaller contribution of fine-grained sediments from the coastal drift and is sufficiently hydrodynamic to prevent the accumulation of silt and very fine to fine sand. On the other hand, the finer particles that are episodically deposited at this depth, are easily resuspended by vortices generated by the bottom forms present in these deposits or in the course of storms [49,52,101–103]. In this region, the DAs may be at least partly removed by waves and transported to other areas by longshore, rip and tidal currents, though in quieter and more favorable periods, the area could be recolonized by living organisms. Thus, the low densities of empty tests and relatively high densities of living organisms found in some stations of the mid ACS (B4-2, B2-2, B2-3) indicate that these areas have been recently affected by stronger hydrodynamic events, probably in the previous winter season, during which the oceanic hydrodynamism is in general more energetic at the Iberian Margin. During winter, there are southwest storms that give rise to downwelling events favoring the transport of material from shallower areas of the continental shelf to deeper zones [99,105–109]. This process associated with tests dissolution/disintegration due to bioerosion or other processes should explain the low density of DAs in the mid shelf gravel deposits. The stronger hydrodynamic events that probably remove fine-grained sediments and foraminiferal tests once succeeded by stable and suitable environmental conditions lead to the development of large living foraminiferal communities.

**Fig 18:**
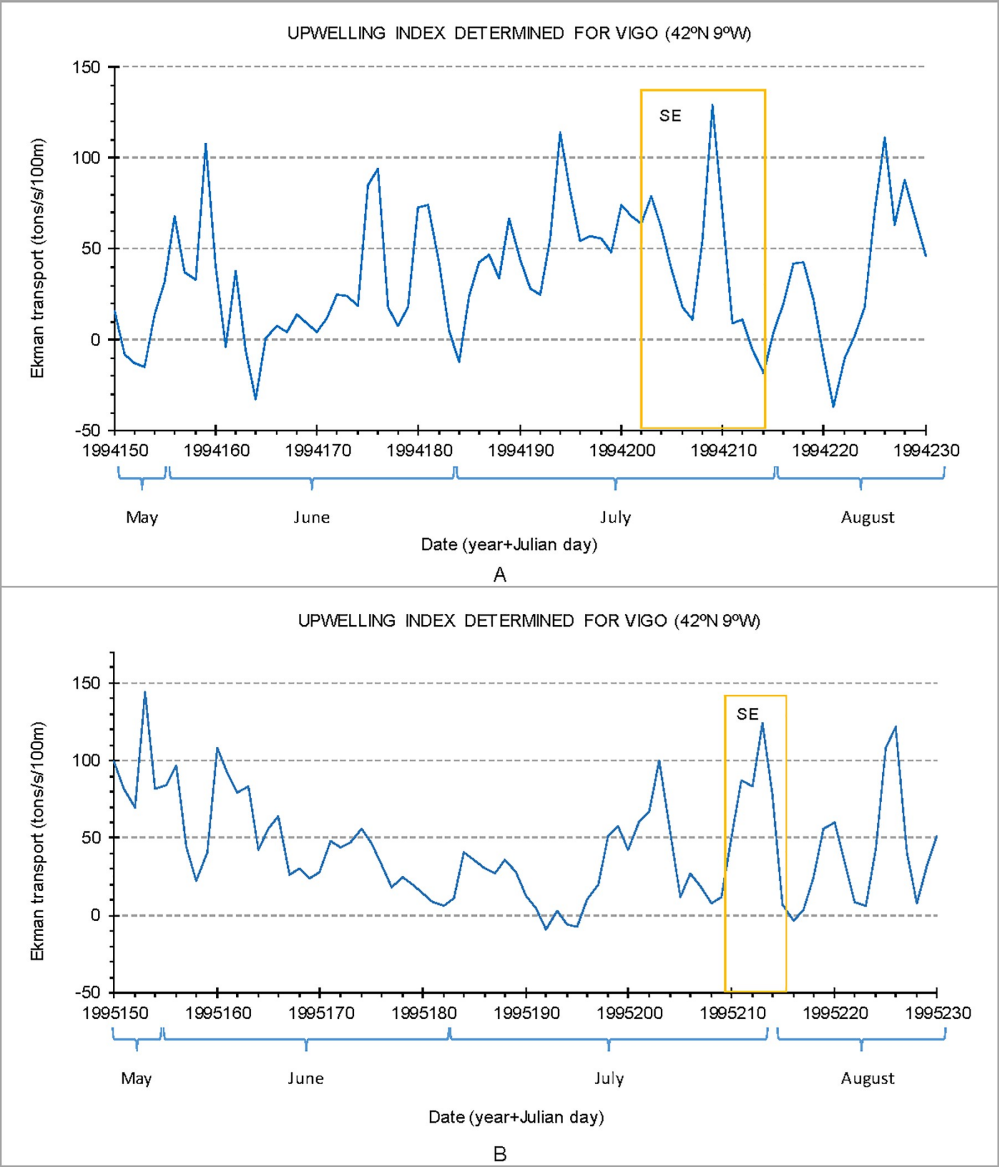
Daily upwelling index determined for Vigo (42°N, 9°W) for three months before and during the sampling events in: A. 1994 and; B. 1995. Legend: SE–sampling events of this work (reprinted from Martins et al. [58]).

The most abundant living species at the gravely deposits zone between 40–60 m, *B*. *ordinaria*, *R*. *auberii* (both abundant) and *G*. *gordialis*, seems to have an opportunistic behavior. After a period of disturbance caused by high hydrodynamism (in the winter time before the sampling events), they were able to rapidly increase the standing crop taking advantage of the occurrence of calmer conditions, high sediment stability and the presence of abundant food. During the three months prior to the sampling and during the field works carried out in 1994 for sampling the stations of transects A, the upwelling index values for Vigo (42°N, 9°W; N Spain) varied significantly (Fig 18). The sampling period was preceded by several strong upwelling events and there was also an event during the field works.

Upwelling events at the Iberian margin trigger the increase of the oceanic productivity and the development of the marine food chains [110–111]. Therefore, the relatively high abundance of living foraminifera at stations between 20–80 m in A-transects should have been favored by the flux of organic matter from oceanic productivity with high quality for benthic organisms, following repeated and significant upwelling events, according to data presented by Martins et al. [58] in addition to that supplied by the Aveiro Lagoon. In this sector, however, the values of TOM content are not the highest of the study area. The high densities of benthic living foraminifera and probably of other organisms contribute for the declining of TOM values due to an intense heterotrophic activity in addition to hydrodynamic conditions, which avoid the accumulation of high amount of organic matter and fine-grained sediments at these depths.

The “receiving” is higher than the “accordance” mostly in the area under the influence of the lagoon outflow due to the opportunistic character of the foraminifera that inhabit the region, which rapidly recovers when the environmental conditions are favorable, also taking advantage of nutrients and organic matter provided by the lagoon to the oceanic system. These results are quite interesting since they evidence that DAs at the time of the sampling are quite different of the LAs, in this region. Bottom hydrodynamic conditions both in the present and in the past, due to the Quaternary evolution of the western Iberian Margin [49,58], seem to directly or indirectly influence the sediments grain-size and the similarity between LAs and DAs densities of the gravel deposits of ACS. In fact, the mean density of living and dead individuals of the 35 most abundant species analyzed by CCA (Fig 12), evidences that the LAs at 0–20 m, 20–40 m and 40–60 m depths intervals are successively associated with coarser sediments while those that are found at depths of 60–100 m, 100–150 m and 150–200 m occur in finer sediments. These results indicate that in ACS, the grain-size of the sediment itself is not a conditioning factor for the development of large communities of living foraminifera, as was also observed by Diz et al. [112] in the Ria de Vigo. Indeed, large and diversified LAs are found at the region where the substrate is composed by gravel deposits in ACS.

#### LAs and DAs at the outer shelf and shelf break

The sedimentary coverture of the outer continental shelf and shelf break consists mainly of medium to very fine sand [51]. However, mostly in the southern sector, there is a well-defined zone of relic deposits possibly originated during the last glaciation, under favorable paleoenvironmental conditions [113]. These relic deposits are composed by coarse sand with significant amount of gravel particles essentially with biogenic origin consisting of fragments of mollusk shells (fossils) and high abundance of foraminiferal tests (a mixture of fossils and recent material [49,50,53]. The sand and gravel fractions of this zone, have low occurrence of detrital grains and significant amount of glauconite [53]. Thus, they have a quite different composition of the gravel deposits of the mid shelf and also have in general smaller mean particle size.

The increase of dead foraminiferal density in deeper continental shelf zones as well as the general increase of fine-grained sediment and TOM (Fig 3A–3C) should be related to the prevalence of relatively low hydrodynamic bottom activity. These relatively low hydrodynamic conditions allow the deposition of fine materials with low density (detrital and particulate OM) and the accumulation of empty tests of foraminifera. However, the sedimentation rate in this region is low as indicate the presence of abundant authigenic glaucony grains in the sediments of this area [53]. In fact, Martins et al. [114] based on ^210^Pb and ^137^Cs, estimated sedimentation rates for the outer continental shelf at northern of this region in 0.28±0.02 cm yr^-1^ and 0.29±0.02 cm yr^-1^, respectively. These values are similar to those obtained by Jouanneau et al. [115] at the NW Iberian continental shelf. Accordingly, the low supply of sediments to the deeper continental shelf areas contributes to the “non-dilution” of empty foraminiferal tests by terrigenous particles [49–52,56]. The pattern of reduction of sediment accumulation rate with depth is however not constant. For instance, on the continental slope adjacent to the Aveiro Canyon, Carvalho and Ramos [116] estimated at 2,800 m and 2,860 m depth, accumulation rates of 0.48 g.cm^-2^.yr^-1^ and 0.1–0.2 g.cm^-2^.yr^-1^, respectively, which evidence the accumulation of abundant material, most likely from the continental shelf break. Although data on the sediment accumulation rate in the region are very scarce for a detailed analysis, the presence of glaucony in the sediments of the outer shelf and shelf break indicates that the bottom of this region receives high supply of organic matter and low supply of terrigenous sediments [49].

Rank distances reaches the highest values again in deeper stations of the outer continental shelf where the IncorpVals decrease to zero (Fig 5). The SimDivers also drop to zero at deeper stations, where living individuals are rare or absent (Fig 7A and 7B). Despite the relatively high TOM contents (1.5–4.5%), in these stations, only a very small number of living organisms are found (Fig 3B).

The plots of Beta diversity comparing LAs and DAs of transect sites evidence that the turnover rates in species number (^0^D) and the turnover in number of dominant species (^1^D) present the same trend of increase with depth (Fig 11A and 11B). This turnover is not due to LAs species or dominant species numbers, which decrease with depth (Fig 11C and 11D) but is determined by DAs, as indicated by plots of Beta diversity (Fig 11E and 11F). The results of the nMDS based on the Diversity/Heterogeneity diagrams (Fig 10), clearly show that such variations occur as depth and TOM contents increase. In the outer continental shelf and shelf break region, the accumulation and preservation of DAs seem to be better than in other sectors of ACS. Thus, considering the negative correlation between TOM and density as well as the reduced diversity in LAs at deeper stations (80–200 m) of transects B1, B2 and B3 and the opportunistic behavior of many foraminiferal species in DAs, such as *U*. *peregrina*, *B*. *spathulata*, *B*. *marginata*, *H*. *boueana*, *B*. *striatula*, *B*. *striata*, *B*. *dilatata* and *N*. *fabum* (Group 2 in the CCA Fig 12) and *B*. *difformis*, *C*. *laevigata*, *G*. *subglobosa*, *G*. *minuta*, *F*. *orbignyana* and *T*. *sagittula* (Group 3 in the CCA, Fig 12), can be deduced that a record of past standing crops of benthic foraminifera was basically found in this region. Increase of DAs densities (Fig 3B) in the outer shelf and shelf break was therefore related to a memory of past standing crops of living benthic foraminifera. Most of these species are indicative of high oceanic productivity related to the upwelling events in the Iberian margin [56].

The export flux of particulate organic carbon (POC) is seasonally and spatially quite variable at the Iberian Basin [117]. Variations between 16–210 mmol C m^-2^ d^-1^ of POC were observed, with the highest values recorded during significant bloom events of diatoms and coccolithophores [118]. Temporal variations were also documented in the time-series of phytoplankton community composition [118]. Elevated fluxes of POC are, in general, observed in summer and low and sporadic fluxes in winter [119–120]. Differences in particle export are significant. Summer is the most dynamic period when compared to winter [117] due to the occurrence of upwelling events [26,38,121]. As described by Martins et al. [58], the upwelling pattern in 1995 did not favor the supply of fresh organic matter to the deeper stations of the study area during the sampling time. It is also possible that the vortices generated by the presence of rocky outcrops on the mid shelf [104,121] blocked the export and deposition of fresh organic matter produced in shallower areas of the continental shelf to the outer sector, as well as fine grained sediments. Considering these results, it can be hypothesized that the relatively high levels of TOM in the outer continental shelf were related to degraded organic matter with poor quality that conditioned the development of large LAs of benthic foraminifera in the outer shelf and shelf break. So, the DAs in this region are simply more time-averaged and thus include a higher beta diversity than LAs that instead are quite reduced.

The fidelity with which time-averaged DAs capture variation in species composition and diversity partitioning of living communities remains a quite interesting issue that has been studied and discussed by Tomašových and Kidwell [4,122] for mollusks. Because foraminifera have a short life cycle and some species are r-strategists and can increase greatly in number in a short period of time, the rate of integration of living foraminifera tests in the DAs is expected to be rapid. So, we may deduce that relatively stable and suitable physicochemical conditions associated to the substrate stability and availability of food of high quality are more important factors than a substrate composed by stable fine-grained sediment enriched in organic matter of poor quality to promote the development of large LAs of benthic foraminifera. However, the memory of the LAs can be lost by erosion, by tests degradation or other effects [123]. These effects in the mid shelf cause a temporal deviation between the LAs and DAs of foraminifera. This deviation is much more pronounced in the inner shelf where the energy of the waves and the currents induce very dynamic sedimentary processes. Thus, it can be considered that in the studied area the best registry of time-averaged DAs can be found in the outer continental shelf.

## Conclusion

The innovative statistical methods presented in this work allowed us to compare LAs and DAs. Results evidence that in most of the stations, the density of DAs is higher than the LAs. Densities of DAs increase, in general, towards the deeper continental shelf zones correlated with an increase in fine-grained sediment and TOM due to reduced removal of foraminiferal tests by hydrodynamic activity and low ‘dilution’ of tests by terrigenous inputs at these depths. The intense processes of sedimentary dynamic and the instability of physicochemical parameters are other unfavorable factors for the development of large living foraminiferal communities in the shallower sector of ACS. In this study, the low density of living foraminifera is found in shallower stations under the influence of the continental drift, due to sediments instability, and in the stations of the outer continental shelf and shelf break probably because of the lack of food quality, conditioned by the occurrence of weak upwelling events during months preceding the sampling campaigns. Densities of LAs are higher between 30 and 50 m at the gravel deposits of the mid shelf. The nMDS results suggest that the LAs and DAs diversity and heterogeneity are similar and almost coincident in coarse grain size sediments of the mid shelf. The stability of the substrate coupled with availability of organic matter from marine productivity and supplied by the Aveiro Lagoon, allow the development of large LAs in this region. However, in some stations a significant loss of DAs in the sedimentary record is observed that might be ascribed to remobilization, transport and dispersion of tests by hydrodynamic forces. With increase of TOM and depth, DAs exhibit high species richness with small variation in heterogeneities, whereas the LAs show reduced diversity and increased heterogeneity as the density of the specimens drops to zero. The upwelling pattern does not favor the development of the LAs of foraminifera in the deeper areas of the PCA, despite the increase in the amount of TOM, probably because of its poor quality. The DAs and LAs of benthic foraminifera are quite different in the ACS induced by oceanographic processes including sedimentary dynamics and productivity caused by upwelling and the Aveiro lagoon outflow; however, the region that seems to best preserve the memory of past standing crops of LAs is the outer continental shelf.

## Supporting information

S1 Table Samples location. Total Organic Matter (%: TOM) and Sediments Granulometry Description. Legend: SMGS—Sediment Mean Grain size. Data from Martins et al. [58].
(XLSX)

S2 Table Density of living and dead foraminifera (n° / 10 cm^3^ of sediment) in the studied samples (SM 1). (XLSX)

S3 Table Environmental variables and assemblage indices of the transect sites. Chao1 and Evenness in standardized form.
(TIF)

S4 Table Beta diversities expressed in Hill numbers between living and dead assemblages and between succeeding transect sites regarding living and dead assemblages separately. (TIF)

S1 Fig Vertical profile of the water column at the stations of transept B 2. of water column: A. temperature (°C); B. salinity and; C. density. Reprinted from Martins et al. [58].
(DOCX)
