# Supplementary material for: Dissimilarity between living and dead benthic foraminiferal assemblages in the Aveiro Continental Shelf (Portugal)
Source: PLoS One. 2019 Jan 30;14(1):e0209066. doi: 10.1371/journal.pone.0209066 (PMC6353080; doi:10.1371/journal.pone.0209066)
Supplement: S1 Fig — Vertical profile of the water column at the stations of transept B 2. of water column: A. temperature (°C); B. salinity and; C. density. Reprinted from Martins et al. [58]. (DOCX) [file pone.0209066.s005.docx]

|   A |   B |   C |
| --- | --- | --- |

**Legend:**

X- Distance (Km) from the most offshore station

Y - Depth (m)

The bottom line is represented by the line drawn on the right side of the graph

| Station | Distance (Km) |
| --- | --- |
| B 2-1 | 44.2 |
| B 2-2 | 36.9 |
| B 2-3 | 29.4 |
| B 2-4 | 22.1 |
| B 2-5 | 14.7 |
| B 2-6 | 7.3 |
| B 2-7 | 0.0 |

**S1 Fig.** Vertical profile of the water column at the stations of transept B 2. of water column: A. temperature (ºC); B. salinity and; C. density (reprinted from Martins et al., 2017).
